# Unveiling the affinity–stability relationship in anti-measles virus antibodies: a computational approach for hotspots prediction

**DOI:** 10.3389/fmolb.2023.1302737

**Published:** 2024-03-01

**Authors:** Rimpa Paul, Keisuke Kasahara, Jiei Sasaki, Jorge Fernández Pérez, Ryo Matsunaga, Takao Hashiguchi, Daisuke Kuroda, Kouhei Tsumoto

**Affiliations:** ^1^ Department of Bioengineering, School of Engineering, The University of Tokyo, Tokyo, Japan; ^2^ Research Center of Drug and Vaccine Development, National Institute of Infectious Diseases, Tokyo, Japan; ^3^ Institute for Life and Medical Sciences, Kyoto University, Sakyo-ku, Kyoto, Japan; ^4^ Department of Chemistry and Biotechnology, School of Engineering, The University of Tokyo, Tokyo, Japan; ^5^ The Institute of Medical Science, The University of Tokyo, Tokyo, Japan

**Keywords:** antibody engineering, computer-aided design, measles virus hemagglutinin, hotspots, relative hydropathy, molecular dynamics

## Abstract

Recent years have seen an uptick in the use of computational applications in antibody engineering. These tools have enhanced our ability to predict interactions with antigens and immunogenicity, facilitate humanization, and serve other critical functions. However, several studies highlight the concern of potential trade-offs between antibody affinity and stability in antibody engineering. In this study, we analyzed anti-measles virus antibodies as a case study, to examine the relationship between binding affinity and stability, upon identifying the binding hotspots. We leverage *in silico* tools like Rosetta and FoldX, along with molecular dynamics (MD) simulations, offering a cost-effective alternative to traditional *in vitro* mutagenesis. We introduced a pattern in identifying key residues in pairs, shedding light on hotspots identification. Experimental physicochemical analysis validated the predicted key residues by confirming significant decrease in binding affinity for the high-affinity antibodies to measles virus hemagglutinin. Through the nature of the identified pairs, which represented the relative hydropathy of amino acid side chain, a connection was proposed between affinity and stability. The findings of the study enhance our understanding of the interactions between antibody and measles virus hemagglutinin. Moreover, the implications of the observed correlation between binding affinity and stability extend beyond the field of anti-measles virus antibodies, thereby opening doors for advancements in antibody research.

## 1 Introduction

In recent years, the application of computational methods has expanded significantly in the field of antibody engineering (Kuroda et al., 2012; Fischman and Ofran, 2018; Kuroda and Tsumoto, 2018; 2020; Akbar et al., 2022a; Wilman et al., 2022). The potential applications are vast; however, predicting biophysical properties can be still challenging when crystal structures of neither the antibody itself nor the antigen-antibody complex are available. This lack of binding information further complicates the task of guiding *in silico* antibody engineering. Numerous computational protocols have been developed to facilitate tasks such as affinity maturation, protein aggregation prediction, and stability enhancement. These aim to create biologically superior antibodies and often rely on initial structure predictions through techniques like homology modelling and molecular docking (Weitzner et al., 2017; Cannon et al., 2019; Liang et al., 2021).

Artificial intelligence (AI) technologies have made significant strides in tackling challenges within protein engineering. The advancements in machine learning (ML) and deep learning (DL) have revolutionized antibody research, particularly in areas such as structure prediction, antibody design, and epitope mapping (Jumper et al., 2021; Ripoll et al., 2021; Akbar et al., 2022b; Prihoda et al., 2022; Ruffolo et al., 2022; 2023). The integration of data-driven AI approaches holds immense promise for drug discovery. However, the accuracy and reliability of these AI predictions heavily rely on the quality of the training data. One significant advancement in developing more robust prediction models is the availability of comprehensive antibody libraries, such as the Observed Antibody Space (OAS) (Marks et al., 2021). OAS has played a crucial role in addressing challenges in antibody engineering, such as humanization and immunogenicity prediction (Olsen et al., 2022; Prihoda et al., 2022). Despite these advancements, certain problems, like trade-offs between antibody affinity and stability remains a challenge as it necessitates large-scale experimental data.

Several studies highlight the same concern of potential trade-offs between antibody affinity and stability in antibody engineering (Rabia et al., 2018). However, current approaches have not specifically addressed the exploration of this relationship. Seizing this opportunity, we followed a knowledge-based computational approach that can identify key residues, thereby revealing the intricate interplay between the affinity and stability of an antibody. This approach utilizes standard *in silico* protein engineering tools and focuses on the importance of residues in the complementarity determining regions (CDRs). CDR3 in the heavy and light chains is widely recognized for its critical role in antigen recognition and binding (Kuroda et al., 2008; Kuroda et al., 2009; Weitzner et al., 2015; D’Angelo et al., 2018). In general, other regions such as framework regions (FRs) in variable domain (Fv) and constant domains primarily contribute to antibody stability (Ionescu et al., 2008; Zabetakis et al., 2013). Nevertheless, we hypothesize that CDR3 residues also contribute to stability and could impact both affinity and stability. To substantiate this, we identified hotspots as sequential pair located within CDRs (particularly focusing on CDR3), by integrating MD simulations to *in silico* alanine scanning. These hotspots are capable of modulating both affinity and stability based on their local or relative hydropathy (Di Rienzo et al., 2021). Relative hydropathy is based on the surroundings of an amino acid side chain, which plays a crucial role in antigen binding and stability.

As a model system, we choose antibodies against the measles virus hemagglutinin (MVH). Measles is an infectious and highly contagious disease that continues to thrive in developing countries, despite the availability of an effective vaccine for decades (Suvvari et al., 2023). To fully eradicate the disease, there is an urgent need for advanced measles therapy. Although researchers have been developing antibodies against measles virus for epitope identification and other research purposes, none of these antibodies have yet entered clinical trials. Remarkably, no crystal structures for anti-measles virus antibodies or antibody-antigen complexes are available in the Protein Data Bank (PDB) (Berman et al., 2007). This lack of structural data is a significant hurdle to the development of antibody-based treatments against the measles virus. On the other hand, the crystal structures of the MVH (Hashiguchi et al., 2007) and a fusion protein, two glycoproteins present in the virus’s envelope, are available in PDB in both apo and holo forms with cellular receptors such as signaling lymphocytic activation molecule (SLAM) (PDB ID: 3ALW, 3ALZ, 3ALX), Nectin-4 (PDB ID: 4GJT), and CD46 (PDB ID: 3INB) (Santiago et al., 2010; Hashiguchi et al., 2011; Zhang et al., 2013). This disparity makes antibodies against measles virus an intriguing subject for further research. In this context, Tadokoro and colleagues (Tadokoro et al., 2020) have extensively analyzed biophysical parameters such as equilibrium dissociation constant (*K*_D_) or binding affinity, melting point *T*_m_ or thermal stability, and thermodynamic parameters for an anti-MVH antibody 2F4. The reported binding affinity for antibody 2F4 Fab at 25 °C was 18 nM, which is about 10 and 37-fold higher affinity than SLAM (*K*_D_ = 170 nM) and Nectin-4 (*K*_D_ = 670 nM), respectively. Neutralization of the virus by the antibody 2F4 has also been reported, along with three other antibodies, namely, 7C6, 8F6, and 10B5 (Sato et al., 2018). All the antibodies obtained from mouse immunization can neutralize the antigen MVH, differing to some extent in the neutralizing capability. These four antibodies have different germline origins (Supplementary Table S1).

In this study, based on homology modeling, docking simulations, MD simulations, and *in silico* alanine scanning, we computationally predicted residues that potentially coupled both stability and binding affinity, and experimentally analyzed physicochemical properties of anti-MVH antibodies. The antibodies we employed demonstrated high binding affinities less than 1 nM to MVH, but they differed in stability. Pairwise point mutational analysis offered insights into these differences and suggested a potential relationship between affinity and stability of anti-MVH antibodies.

## 2 Results

### 2.1 Experimental characterization of anti-measles virus neutralizing antibodies

We first performed physicochemical analysis of the four wild type (WT) antibodies: 2F4, 7C6, 8F6, and 10B5. These antibodies were previously obtained through mouse immunization (Sato et al., 2018) and, except for 2F4 (Tadokoro et al., 2020), they had not been biophysically characterized until this study. Ideally, antibodies should demonstrate a rapid association and a slow dissociation with antigens. Our SPR measurements confirmed that antibodies 7C6 and 8F6 exhibited these characteristics, resulting in an affinity of 0.4 ± 0.2 and 0.9 ± 0.2 nM, respectively, toward MVH (Table 1). On the other hand, 2F4 and 10B5 demonstrated a slower association and a faster dissociation, resulting in lower binding affinity of 54.1 ± 0.1 and 60.3 ± 19.4 nM, respectively.

**TABLE 1:** Physicochemical analysis of the wild type anti-MVH antibodies. Kinetic parameters^a^ and melting temperature (*T*_m_) are shown.

Physicochemical analysis (wild type)		< 1 nM affinity group		> 50 nM affinity group	
		**7C6**	**8F6**	**2F4**	**10B5**
Binding affinity	*k*_on_ (×10^5^ M^−1^s^−1^)	11.4 ± 4.8	3.4 ± 2.9	1.2 ± 0.5	0.1 ± 0.1
	*k*_off_ (×10^−4^ s^−1^)	4.1 ± 1.7	2.8 ± 1.7	65.8 ± 29.1	8.6 ± 0.4
	*K*_D_ at 25°C (nM)	0.4 ± 0.2	0.9 ± 0.2	54.1 ± 0.1	60.3 ± 19.4
Thermal stability	*T*_m_ (°C)	73.9 ± 0.3	68.0 ± 0.1	72.7 ± 0.1	73.9 ± 0.1

^a^ The simple 1:1 Langmuir binding model was used to fit and calculate the kinetic parameters of the binding.

The *K*_D_ of 2F4 antibody reported in a previous study (Tadokoro et al., 2020) was lower than our observed value (Table 1; Supplementary Figure S1). Despite this discrepancy, all four antibodies exhibited better binding affinity than the receptors, particularly 7C6 and 8F6. Although the reported thermal stability of the 2F4 Fab was 76°C (Tadokoro et al., 2020), our DSC measurements revealed a decrease in melting temperature (*T*_m_ = 72.7°C ± 0.1°C). Antibodies 7C6 and 10B5 demonstrated higher stability with melting temperatures of 73.9°C ± 0.3°C and 73.9°C ± 0.1°C, respectively, while 8F6 exhibited lower thermal stability of 68.0°C ± 0.1°C (Table 1; Supplementary Figure S2).

Based on these observations, we classified the antibodies into two affinity groups (Table 1). Subsequently, we focused on the high binding affinity (<1 nM) antibodies 7C6 and 8F6, which showed a significant difference in thermal stability (*ΔT*_m_, ∼6°C). Analyzing these characteristics may provide insights into the relationship between binding affinity and thermal stability in anti-MVH antibodies.

### 2.2 Homology modeling and antibody-antigen local docking

**FIGURE 1:**
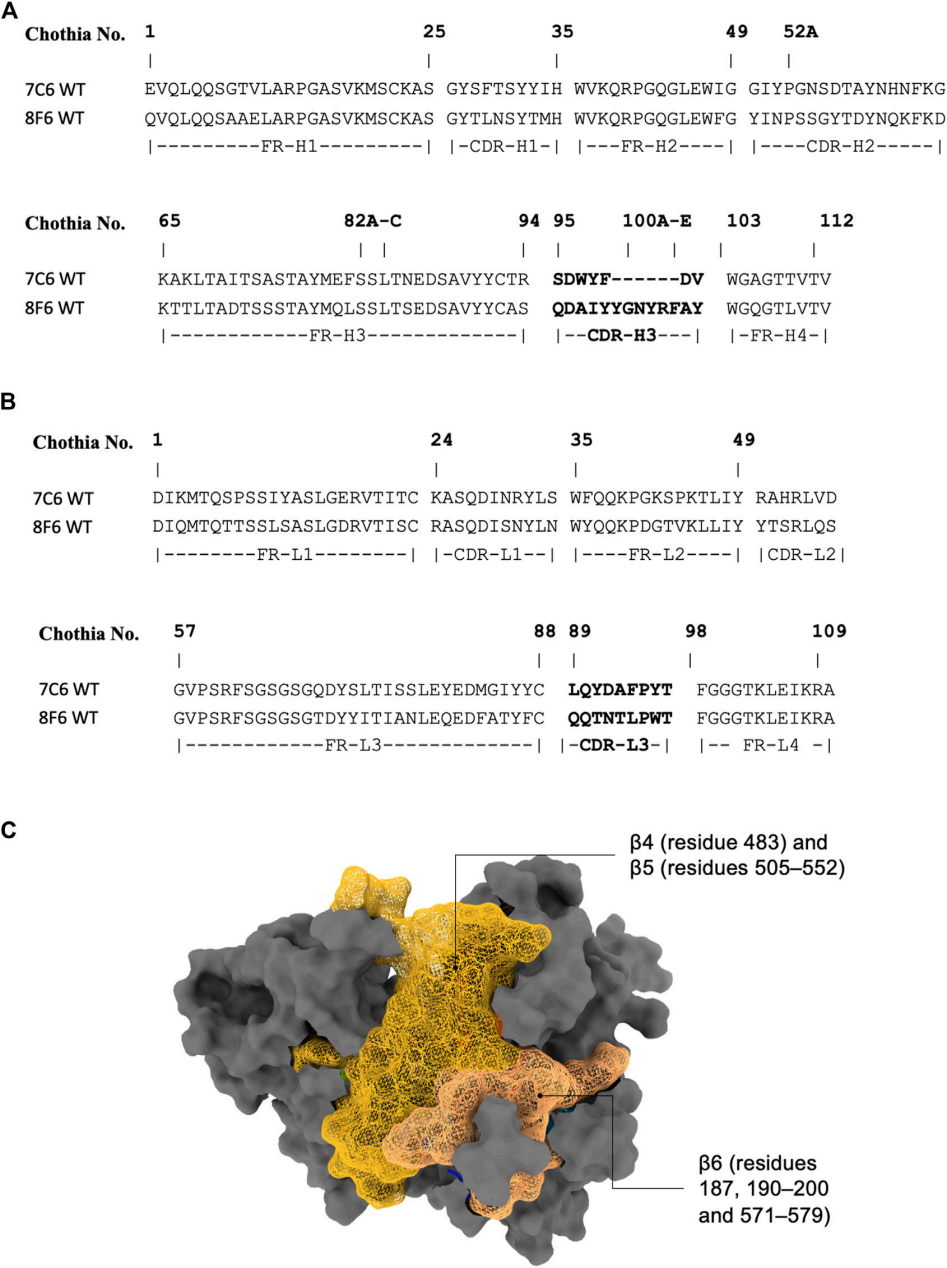
Antibody sequence and epitope of MVH. **(A)** and **(B)** display the antibody sequence of the heavy and light chains, respectively. **(C)** illustrates the head domain of the measles hemagglutinin, showcasing the epitope used in this study represented as a mesh-like surface. The non-epitope region is colored gray.

As the crystal structure of the antibodies are unavailable at the time of this writing, we performed antibody structure modeling with the RosettaAntibody protocol (Weitzner et al., 2017). The variable fragment of the antibody was modeled from the amino acid sequences (Figures 1A, B), and the best scored model was selected for docking with the MVH crystal structure (PDB ID: 2ZB6). While there was no prior binding information available for the high-affinity antibodies (7C6 and 8F6), it was available for the receptors. The head domain of the MVH has 6-bladed β-propeller folds (β1–6). It is the main target of neutralizing antibodies (Tahara et al., 2016). Among them, the receptor binding epitope, which is a group of amino acids in the receptor binding site, stands out because, as the name suggests, it is also recognized by the three receptors to MVH, as well as by antibody 2F4. It is worth noting that several other antibodies, which were not included in this study, have also been reported to target this epitope (Tahara et al., 2016). The receptor binding epitope is located primarily within β5 with some extension in β4 and β6. Since 2F4 is reported to interact with the receptor binding epitope (Tahara et al., 2016), we first constructed a putative structure of the 2F4 with MVH by placing the antibody within 7 Å of the MVH near the receptor binding epitope, so that the CDRs and the receptor binding epitope roughly face each other. Next, we performed a Monte Carlo-based rigid body docking using RosettaDock (Chaudhury et al., 2011), that predicted favorable binding modes of 2F4 with MVH. The best docking score obtained was −26.9 Rosetta Energy Unit (REU). The visual inspection of this docked model showed that amino acids 190, 533 and 541, which reported to recognize 2F4 is within 5 Å, in agreement with the reported experimental data (Tahara et al., 2013; 2016). The 2F4 docked model helped in our knowledge-based docking approach and we used it as a reference to construct the putative model for 7C6 and 8F6 followed by flexible antibody-antigen docking (Weitzner et al., 2017). The “core epitope” utilized in this study encompasses the following amino acids in the receptor binding site of MVH: 187, 190–200 and 571–579 in β6, 483 in β4, 505–552 in β5 (Figure 1C). Binding of antibodies to this core epitope could identify key interacting residues.

**FIGURE 2:**
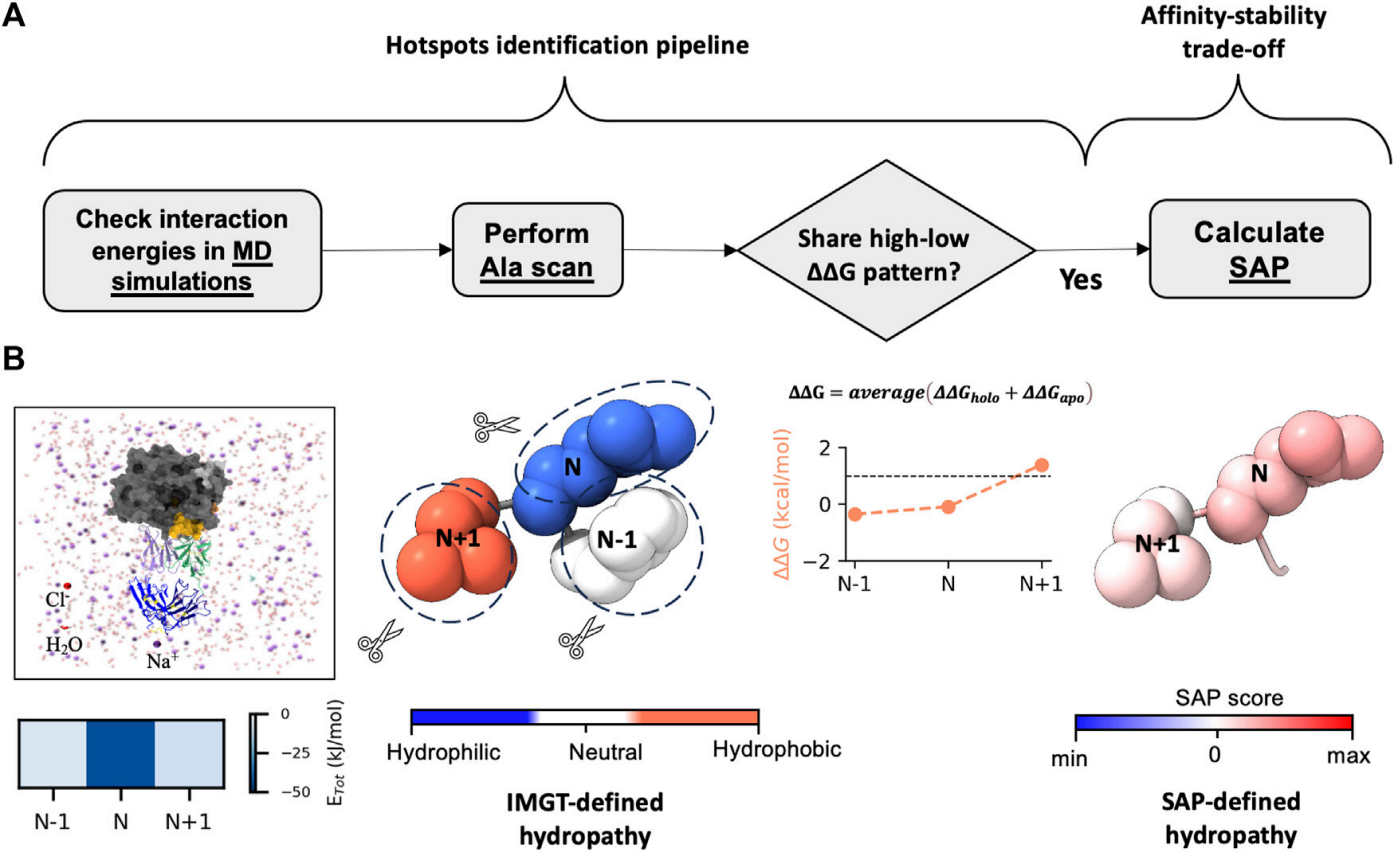
Workflow of hotspots identification pipeline. **(A, B)** display the steps for hotspots identification and their link to affinity-stability trade-offs. **(A)** presents the workflow of the *in silico* experiments, which involves checking the interaction energies of the complex structure by using MD simulations. We identify the residue N exerting strongest interaction energy (total non-bonded interaction energy (E_Tot_)). We then perform *in silico* alanine scan (Ala scan) on the structures (both apo and holo forms). After averaging the ΔΔ*G* from both apo and holo structures, we check whether residue N shares a high-low ΔΔ*G* pattern with its neighbor residues N-1 and N+1. If it does, we pair the residues and predict the pair as hotspots. Finally, we check the relative hydropathy of the pair by calculating spatial aggregation propensity (SAP). This step helps in understanding the interplay between affinity-stability trade-offs. **(B)** illustrates the workflow shown in **(A)**. The residues are displayed as atoms in sphere style, with color coding based on the IMGT-defined hydropathy and SAP score.

Subsequently, with the SnugDock algorithm (Sircar and Gray, 2010), we obtained the best docking scores of −41 REU and −39.5 REU for 7C6 and 8F6 antibodies, respectively. The order of these docking scores aligns with the experimental *K*_D_ values (0.4 ± 0.2 and 0.9 ± 0.2 nM for 7C6 and 8F6, respectively). We also employed docking local refinement in Rosetta to compute the docking score for the available crystal structure of the receptor-antigen complex as a positive control. The best docking scores for receptors SLAM (PDB ID: 3ALZ) (Hashiguchi et al., 2011), CD46 (PDB ID: 3INB) (Santiago et al., 2010) and Necin-4 (PDB ID: 4GJT) (Zhang et al., 2013) were −38.9, −33.7 and −30.9 REU, respectively. These docking scores are aligned well with the reported experimental binding affinity (*K*_D_ 170, 200 and 670 nM for SLAM, CD46 and Nectin-4, respectively) (Hashiguchi et al., 2007; Santiago et al., 2010). The resulting models for antibodies, representing the predicted holo form, were then further evaluated through *in silico* and *in vitro* assessments. The workflow for the *in silico* assessments is depicted in Figure 2.

### 2.3 Visual inspection and MD simulations to identify interacting residues in predicted complex structures

In line with our proposed workflow for hotspot prediction (Figure 2), our initial step involves identifying the interface residues contributing to binding between the antibody and the core epitope. To achieve this, we performed interface analysis of the predicted holo form using UCSF Chimera (Pettersen et al., 2004). We considered residues within a 5 Å distance from both the core epitope and the antibody as interface residues. Among the interface residues identified for 7C6, 46 residues were found in MVH, with 62.8% of them belonging to the core epitope region. In contrast, a total of 31 residues were identified in the antibody as interacting residues (13 and 18 residues in the heavy and light chains, respectively). Notably, all of the identified interface residues in the heavy chain and 77.8% in the light chain were located within the CDRs. Throughout this study, we followed Chothia numbering scheme (Chothia and Lesk, 1987; Al-Lazikani et al., 1997) to define CDRs (Figures 1A, B). Moving on to the 8F6 antibody, we identified 36 interacting residues in MVH, with 74.4% of them belonging to the core epitope region. Additionally, we found 27 interacting residues in the 8F6 antibody, out of which 18 were in the heavy chain, and all of them situated within the CDRs. From this analysis, we deduced that the CDRs of the heavy chain exhibited a reasonable number of interacting residues in the antibodies, particularly in the case of 8F6. Notably, the light chain of 7C6 exhibited a higher presence of interfacial residues than the heavy chain, emphasizing its importance in the interactions.

To computationally assess the validity of the predicted interacting residues of the antibody-antigen complexes, we employed MD simulations. In MD simulations, model structures are refined as they interact with surrounding explicit water molecules. This makes MD simulations a common tool for refining model structures (Heo et al., 2021). To confirm the quality of the simulations we first checked convergence of the three independent MD simulations for each antibody-antigen complex. The convergence of the predicted complex is difficult to achieve since the crystal structure of the MVH (PDB ID: 2ZB6) we used in our docking simulations has missing residues (167–183 and 240–246) in the non-epitope region (Figure 1C). Therefore, we trimmed the terminals of MVH and repaired the missing residues 240–246 through Modeller (Fiser et al., 2000; Webb and Sali, 2016) before the MD simulations. In addition, we modeled the constant regions of the antibody to mimic the Fab format used in experiments. The contribution of the modeled regions was evident in the simulation runs which caused the higher structural deviations in the trajectories. Given that our above interface analysis of the docked models indicated that the interacting residues were primarily located in the core epitope, we focused our attention on verifying the potential interactions within the core epitope and Fv of antibody. Therefore, we checked the convergence using the root mean square deviation (RMSD) of the Cα atoms for these regions, which remained quite stable after 170 ns (Supplementary Figure S3). We used the last 70 ns of the trajectories after achieving convergence in the analyses below.

**FIGURE 3:**
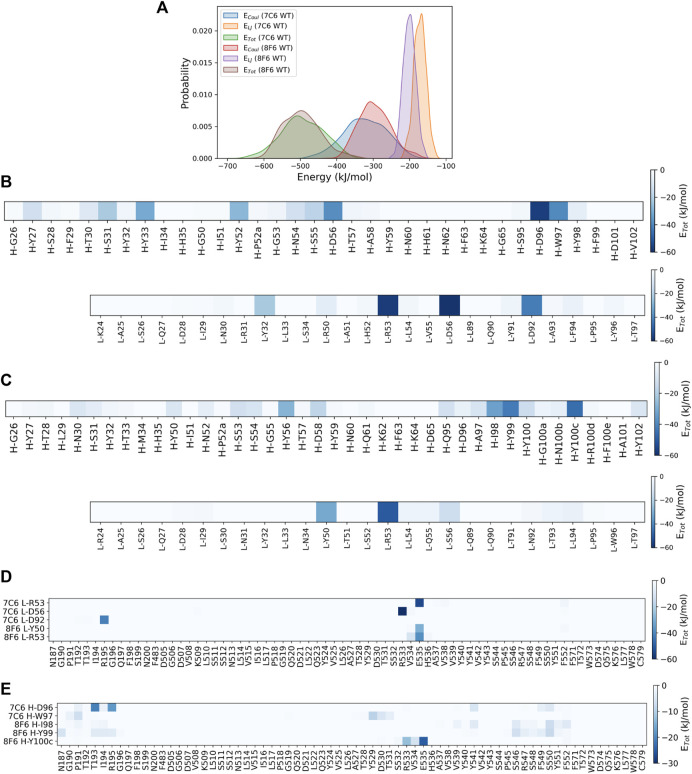
Identifying interacting residues by MD simulations. **(A)** probability distribution functions for interaction energies between antibody and epitope. The data shown for the average of three independent MD simulations. The total non-bonded interaction energy (E_Tot_) shown in kJ/mol, E_Tot_ = Coulombic energy (Coul) + Lennard-Jones (LJ) energy. **(B, C)** display the heatmaps of residue-wise E_Tot_ between the epitope and CDRs of 7C6 and 8F6, respectively. Interaction energies for heavy chain and light chain CDR residues are shown. **(D, E)** present heatmaps of residue-wise E_Tot_ between the CDR and the epitope. These figures illustrate a quasi-epitope mapping for CDR-L and CDR-H residues with largest interaction energies shown in Figure 3BC, respectively.

To identify the residue-wise contributions of interactions between antibody CDRs and the core epitope more quantitatively, we computed the interaction energies (comprising van der Waals and coulomb energy) based on the MD trajectories (Figure 3). The probability distribution function of the non-bonded energy components for both antibodies showed strong interaction energies toward the core epitope. The well-defined peaks observed in Figure 3A suggest the system was in stable configurations during the interactions. We also calculated the energy contribution from residues in all six CDRs (Figure 3BC). The total interaction energy observed for CDRs of 7C6 was −488.5 kJ/mol (H-CDRs: −285.4 kJ/mol and L-CDRs: −203.1 kJ/mol), which was stronger than the interaction energy of 8F6 CDRs at −409.6 kJ/mol (H-CDRs: −294.7 kJ/mol and L-CDRs: −114.9 kJ/mol), in agreement with our experimental results of SPR (Table 1). On a residue-wise basis, a few L-CDR residues contributed significantly to the interaction energy (Figure 3B), whereas multiple heavy chain residues made notable contributions. For 8F6, a similar energy contribution profile was observed for its H-CDR residues (Figure 3C). It is worth noting that all six CDRs contributed to the interaction energies observed in 7C6. In contrast, for 8F6, L-CDRs appeared to make no discernible contribution to the interaction energies except for CDR-L2. We further calculated the interaction energies between the core epitope residues and the selected CDR residues that exhibited significant interaction energies, as seen in Figures 3A, B. Residue L-R53 in the L-CDR2 of both antibodies demonstrated a pronounced interaction energy with residue E535 of the core epitope (Figure 3D). More core epitope residues interacted with H-CDR residues (Figure 3E) than with L-CDR residues (Figure 3D). Figures 3D, E illustrate a quasi-epitope mapping of the MVH for 7C6 and 8F6 antibodies. The possible binding site of 7C6 and 8F6 could be within β6 (187–195) and β5 (529–535, 541, and 546–552).

### 2.4 *In silico* alanine scanning to identify hotspots for thermal stability and binding affinity

The next step in our proposed workflow (Figure 2) entails confirming the key residues for binding. To achieve this, we performed *in silico* Ala scanning (hereafter Ala scan) using FoldX (Schymkowitz et al., 2005). We employed Ala scan on both apo (antibodies only) and holo (antibody-antigen complexes) forms. We included apo forms in this analysis because the loss of binding may originate from the collapse of the antibody structure itself. Hereafter, we referred to ΔΔ*G* as the average value estimated from the ΔΔ*G* of both the apo and holo forms. We utilized the standard cut-off of ΔΔ*G* ≥ 1 kcal/mol for hotspot prediction in protein engineering (Liu et al., 2011; Peng et al., 2014). Positions with ΔΔ*G* above the cut-off are identified as predicted hotspots. From the ΔΔ*G* profile, we first noticed that hydrophobic residues tend to exhibit higher ΔΔ*G* (Supplementary Figure S4, 5). This is likely because they were buried in the antibody structures or at the antibody-antigen interfaces and mutating such a buried residue to Ala would lead to an unstable structure in the apo and holo forms, respectively.

**FIGURE 4:**
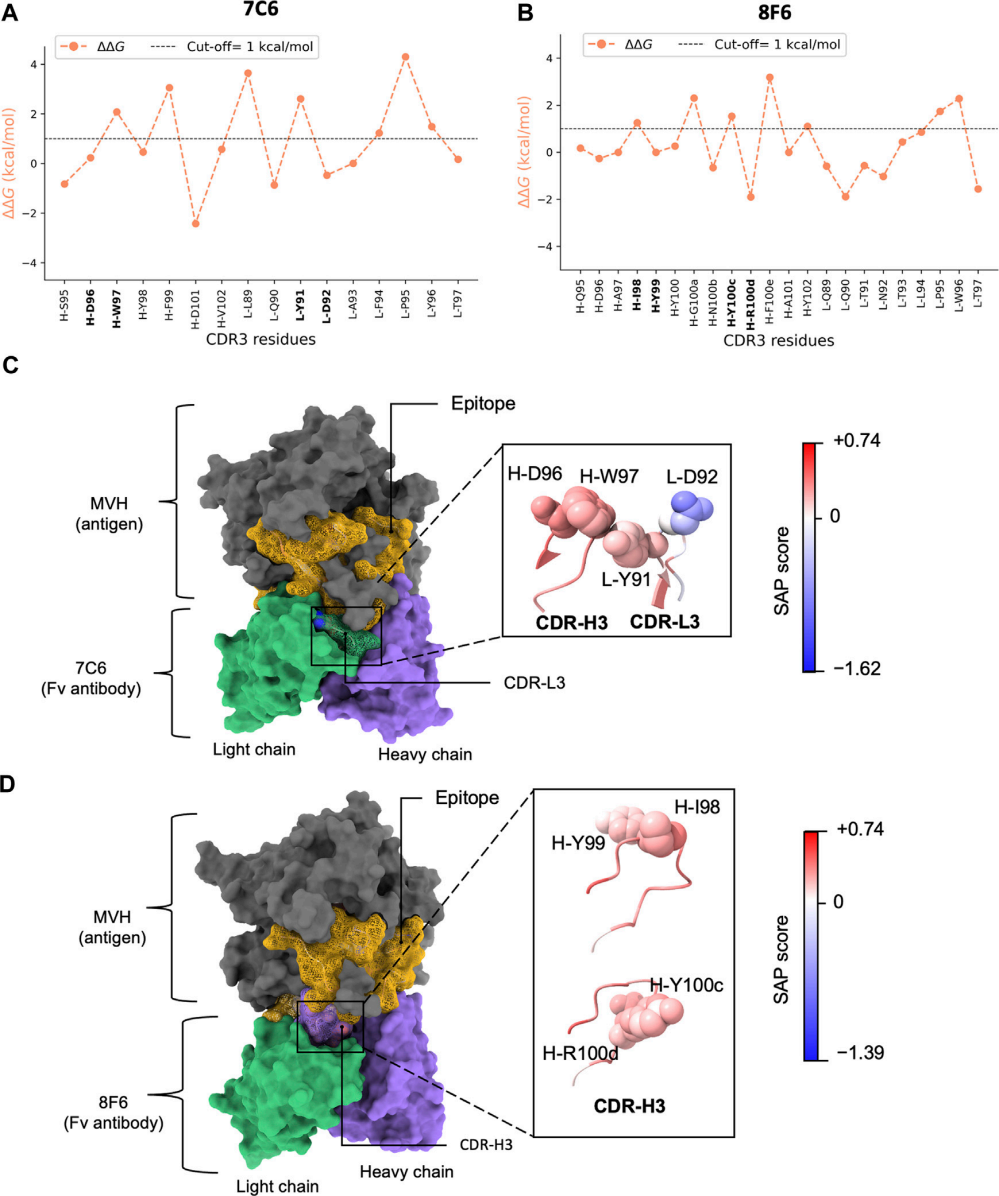
*In silico* alanine scanning and relative hydropathy analysis. **(A, B)** show the results of *in silico* alanine scanning using the FoldX AlaScan command. The results are depicted as an orange line. The ΔΔ*G* cut-off = 1 kcal/mol is represented by dashed line. These plots highlight the four identified residue pairs for antibodies 7C6 (illustrated in **(C)** and 8F6 (illustrated in **(D)**. **(C)**, **D)** display the holo forms of the 7C6 and 8F6 antibodies, respectively. The epitope is represented as a golden mesh-like surface, the non-epitope region is colored in gray, and the heavy and light chains are shown in purple and green, respectively. The CDR3 region is highlighted with a mesh-like surface. The identified residue pairs are displayed as atoms in sphere style, with color coding based on the SAP score. The corresponding SAP scale used for both antibodies is also depicted in the image. The molecular representations were visualized using UCSF ChimeraX.

Second, we also observed a distinct visualization of the high-low ΔΔ*G* pattern (Figure 2; Supplementary Figure S4, 5), which prompted us to further focus on a subset of 2 residues or “pair”. Together with the MD results (Figure 3), we inferred that certain residues paired with its sequential adjacent residues. The sequential pairs for 7C6 were L-R53/L-L54, L-V55/L-D56, L-Y91/L-D92 and H-D96/H-W97 (Supplementary Figure S4). For 8F6, the sequential pairs were L-R53/L-L54, H-I98/H-Y99 and H-Y100c/H-R100d (Supplementary Figure S5). Since our focus of this study is to understand the intricate interplay between binding affinity and stability, we decided to focus on the sequential pairs found in CDR3: L-Y91/L-D92 in 7C6 CDR-L3, H-D96/H-W97 in 7C6 CDR-H3 and H-I98/H-Y99 and H-Y100c/H-R100d in 8F6 CDR-H3 (Figure 4AB). We hypothesized that focusing on the CDR3 region would provide insights into affinity-related trade-offs since, among the CDRs, CDR3 contributes primarily to the binding affinity.

Interestingly, considering the amino acid types, Tyr exhibited a duality nature in the Ala scan depending on the partner residues. When the partner residue is hydrophilic, i.e., Asp (7C6 L-D92) or Arg (8F6 H-R100d), Tyr showed high ΔΔ*G*. On the other hand, when the partner residue is hydrophobic (8F6 H-I98), Tyr showed low ΔΔ*G*. Despite being an aromatic residue, Tyr falls under the “neutral” class of IMGT-defined hydropathy (Pommié et al., 2004), which may explain this duality in the ΔΔ*G* profile.

Thus, from the above analysis, it was suggested that a pattern of high-low ΔΔ*G* observed in this study (Figure 4AB) may be utilized to identify residues in subset or pair that potentially contribute both thermal stability and binding affinity. MD simulation helped in drawing our attention to the residues in CDRs where the pattern is distinct. Even though more favorable interaction energies were observed for L-R53, we chose to focus on the residues in CDR3 that matched our criteria of selection. A high-low ΔΔ*G* pattern shared by the pairs suggested that the hydrophobic partner residues likely aid in interactions by stabilizing the conformation of the partner residues tailored for binding.

### 2.5 Relative hydropathy analysis

The dual hydropathic nature of Tyr prompts questions about its relative hydropathy and its contribution to affinity and stability. To explore this, we investigated the factors that influence change in amino acid hydropathy. We observed non-bonded interactions (van der Waals and coulomb) between antibody and antigen with Coulombic interactions playing a dominant role (Figure 3A). Antibody 7C6 exhibited stronger attractive forces compared to 8F6. The surrounding environment, including water molecules (hydration) in a biological system, influences these interactions. Changes in the environment can alter the chemical nature of an amino acid, affecting the hydrophobic or hydrophilic nature of the amino acid side chain. Recently, Rienzo et al. characterized the hydropathy profiles of amino acid side chains at the protein-solvent interface (Di Rienzo et al., 2021). Inspired by their work, we were prompted to calculate the relative hydropathy of the identified pairs based on their surroundings.

We computed the relative hydropathy on the holo form (Figure 4CD) using spatial aggregation propensity (SAP) (Chennamsetty et al., 2009). SAP identifies hydrophobic patches on a protein’s surface based on a defined radius (R) called SAP radius. Chennamsetty and colleagues (Chennamsetty et al., 2009) reported that hydrophobic interaction plays a key role in protein aggregation, thus impacting stability. A SAP radius of 5 Å could identify the aggregation-prone patches with detailed view. Conversely, a SAP radius of 15 or 20 Å tends to eliminate the hydrophobic patches and favor the hydrophilic patches (Chennamsetty et al., 2010). Thus, to identify the true nature of the amino acid pairs, we employed a SAP radius of 10 Å that could favor both hydrophobic and hydrophilic patches, maintaining a balance between them. We provided a schematic illustration of the alterations in hydropathy in Figure 2B. Upon analyzing the residue pairs in CDR3, we observed pair L-Y91/L-D92 in CDR-L3 of 7C6 (Figure 4C), have a balanced hydrophobic and hydrophilic nature respectively, while the other pairs H-D96/H-W97 in CDR-H3 of 7C6, and H-I98/H-Y99 and H-Y100c/H-R100d in CDR-H3 of 8F6 contributed to the hydrophobic gradient (Figure 4CD). The observation that an IMGT-defined hydrophilic Asp and Arg experiences a distinct change in its hydropathic nature (such as 7C6 H-D96 and 8F6 H-R100d becoming hydrophobic, while 7C6 L-D92 remains hydrophilic) may provide valuable insights into their connection with stability. This is particularly relevant since charged residues are typically not buried without neutralizing their charge, often by forming salt bridges with other residues. Without such compensation, buried charged residues could lead to unstable protein structures. This emphasizes the critical role of the protein environment in considerations of residue hydropathy and its impact on the trade-off between stability and binding affinity.

To further explore the relationship between affinity and stability, and to validate our computational predictions, we subjected the identified paired residues to *in vitro* alanine scanning experiments. This *in vitro* validation is particularly critical given the limited scope of our dataset, comprising only four pairs. Drawing broad conclusions from such a small dataset can be precarious. With this in mind, our experimental validations were designed to assess whether mutations at these positions could alter the characteristics of these pairs, thereby affecting both binding affinity and thermal stability. For the pairs identified in 7C6, which has two types of pairs within CDR3 (Figure 4C), in addition to introducing alanine, we also predicted other amino acid substitutions at the same positions using standard *in silico* tools.

We employed two methods to predict new mutations based on the high-low ΔΔ*G* pattern derived from Ala scan analysis of FoldX. For residues with high ΔΔ*G* values (such as 7C6 L-Y91 and H-W97), which we hypothesized have an impact on stability, we utilized Rosetta’s Cartesian_ddg application on the apo form to predict potential mutations. We chose to use two different methods for ΔΔ*G* calculations–FoldX and Rosetta–because they are orthogonal methods. They utilize distinct rotamer libraries and scoring functions, capturing different aspects of the underlying physics. On the other hand, residues with low ΔΔ*G* values (7C6 L-D92 and H-D96A) suggested that the effects of mutations at these positions are minimal. Therefore, we continued to use FoldX to predict mutations for these residues in both apo and holo forms. Mutations with values below the cut-off (−1 kcal/mol) from the *in silico* mutational analysis were chosen for the *in vitro* mutagenesis study (Supplementary Figure S6). The only exception was for 7C6 H-W97, which did not meet the cut-off. The amino acid Phe was predicted for residues L-Y91, L-D92 and H-D96.

### 2.6 Experimental physicochemical analysis of the antibody mutants

**FIGURE 5:**
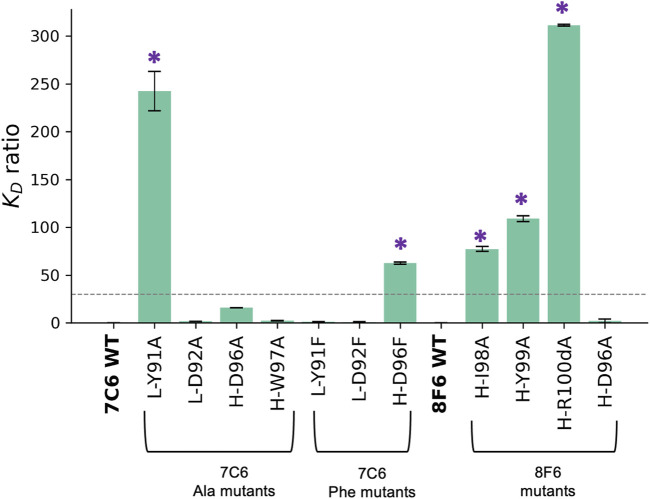
Effect of mutations on binding affinity. Binding affinity is measured by SPR. Effect on binding affinity was measured in terms of *K*_D_ ratio = *K*_D_ of mutant/*K*_D_ of wild type. The wild type (WT) 7C6 and 8F6 antibodies served as the baseline (i.e 0), indicating no change in binding affinity. Error bars were calculated from three independent measurements, and asterisks denote mutants that exhibit a significant change in binding affinity, which corresponds with the > 30-fold decrease in binding affinity (Akiba and Tsumoto, 2015).

We expressed the mutants (Table 2) and purified them using size-exclusion chromatography (SEC). Similar to the WT antibodies, we conducted SPR analysis for the mutants to measure the binding affinity and compared the change in binding affinity or *K*_D_ ratio (Figure 5). For the Ala mutants of the predicted hydrophobic-hydrophobic pairs identified in 8F6 (H-I98/H-Y99 and H-Y100c/H-R100d), significant loss of binding affinity was observed. Ala mutation to H-Y100c exhibited a weak binding to the extent that kinetic fitting was not applicable (Supplementary Figure S7B), revealing that this position is also a hotspot for binding. This suggests that all residues involved in the hydrophobic-hydrophobic pairs of 8F6 were critical for binding. As a control, we chose 8F6 H-D96, which is spatially near the hotspot pair H-Y100c/H-R100d in 8F6 (Supplementary Figure S8). Although H-D96 was not predicted as a hotspot in our approach, its proximity and the charged nature of aspartic acid suggested its potential importance for binding. However, despite its location within the CDR-H3, H-D96 showed a negligible change in binding affinity (Figure 5 and S8). This outcome serves as validation for our hotspot identification pipeline (Figure 2), confirming the accuracy of not identifying this residue as a hotspot.

**TABLE 2:** Kinetic and thermal stability parameters of the 7C6 and 8F6 mutants.

	*k*_on_ (×10^5^ M^−1^s^−1^)	*k*_off_ (×10^–4^ s^−1^)	*K*_D_ at 25°C (nM)	*T*_m_ (°C)	Δ*T*_m_ (°C)
**7C6 WT**	11.4 ± 4.8	4.1 ± 1.7	0.4 ± 0.2	73.9 ± 0.9	
L-Y91A	1.8 ± 0.5	168.8 ± 35.0	97.5 ± 8.2	71.3 ± 0.7	−2.6
L-D92A	7 ± 0.4	4.6 ± 0.4	0.7 ± 0	73.7 ± 1.7	−0.3
H-D96A	14.2 ± 0.3	90.8 ± 0.2	6.4 ± 0.1	75 ± 1.7	1.0
H-W97A	43.6 ± 5.1	37.6 ± 1.6	0.9 ± 0.1	72.9 ± 0.3	−1.0
L-Y91F	27.0 ± 1.0	13.2 ± 0.3	0.5 ± 0	71.7 ± 1.1	−2.2
L-D92F	14.4 ± 0.2	4.4 ± 0.2	0.3 ± 0	72.7^a^	−1.2
H-D96F	14.4 ± 1.3	363.1 ± 27.3	25.2 ± 0.4	72.8 ± 0.8	−1.1
**8F6 WT**	3.4 ± 2.9	2.8 ± 1.7	0.9 ± 0.2	68.4 ± 0.9	
H-I98A	2.2 ± 0.8	153.3 ± 48.8	70.5 ± 2.4	69.1 ± 1.0	0.7
H-Y99A	0.1 ± 0	8.9 ± 0.2	99.5 ± 2.8	68.6 ± 0.4	0.2
H-Y100cA	- -	- -	N.D.	69.7 ± 0.7	1.3
H-R100dA	0.1 ± 0	40.5 ± 0.3	284.0 ± 0.9	70.2 ± 0.4	1.8

N.D., not determined as kinetic fitting was not applicable.

^a^ *T*_m_ measurements for 7C6 L-D92F were conducted only once due to insufficient protein quantity.

For the Ala mutants of 7C6, we identified L-Y91 from the hydrophobic-hydrophilic pair as a key residue with a loss in binding affinity of about 242-fold. On the other hand, its partner residue, L-D92, had no significant effect on binding affinity (Figure 5). In contrast, within the hydrophobic-hydrophobic pair, the H-D96A and H-W97A mutants in CDR-H3 of 7C6 showed a 16-fold loss and a negligible change in binding affinity, respectively. Additionally, the differences in the *K*_D_ ratio between key residues found in CDR-L3 and CDR-H3 suggested that light chain accommodated the primary hotspot. The Ala mutants resulting in reduced binding affinity of the high-affinity antibodies to MVH echoed one common cause of loss of binding, that is faster *k*_off_ (Table 2).

The Phe mutants to 7C6 showed tolerance for Phe mutation at the primary hotspot pair (L-Y91/L-D92), which is consistent with the docking scores (Supplementary Figure S9). Furthermore, the hydropathy of these Phe mutations aligned with the hydropathy of the pair in the WT, suggesting an explanation for the pair’s ability to tolerate the mutations. In contrast, the mutant H-D96F in CDR-H3 showed a 63-fold loss in binding affinity. This suggests that the secondary hotspot is also contributing to the overall binding affinity of 7C6 and did not tolerate a mutation to a bulky residue like Phe.

**FIGURE 6:**
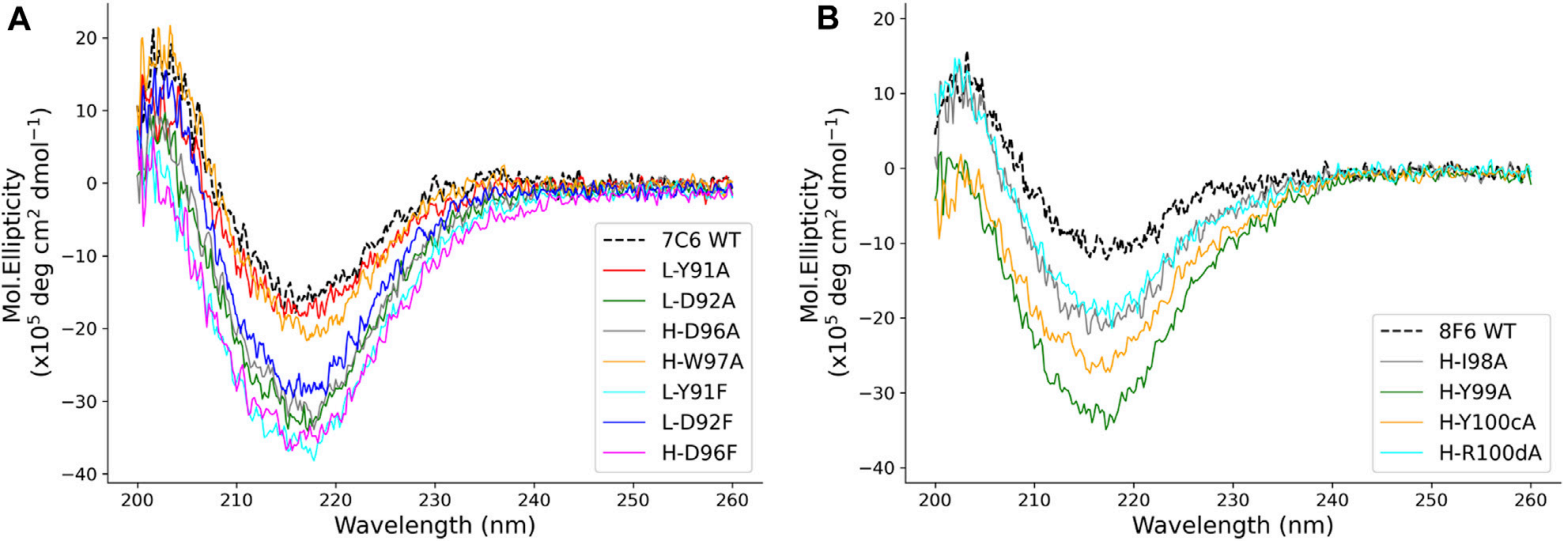
CD profile of the mutants. **(A, B)** show the CD profile of the mutants for 7C6 and 8F6, respectively.

We performed circular dichroism (CD) to observe any structural changes that may have occurred due to the point mutations causing these changes in binding affinity (Figure 6). The CD spectrum for all the mutants retained the beta-sheet like folding that generally observed for Fab antibodies (Cathou et al., 1968). In addition, some changes in molar ellipticity were observed for the mutants, but the results were not conclusive to provide sufficient information about the type of structural changes. Thus, we next performed thermal stability measurements to observe the effect of mutations on the melting temperature (*T*_m_) of the mutant antibodies.

Due to insufficient yield, we employed CD measurements instead of DSC to determine the *T*_m_ of the mutants. The *T*_m_ of WT 7C6 remained consistent in both DSC and CD measurements (*T*_m_ in CD: 73.9°C ± 0.9°C and DSC: 73.9°C ± 0.3°C), while a negligible difference was observed for the 8F6 WT antibody (Δ*T*_m_ ∼0.5°C). Therefore, we used the *T*_m_ obtained from CD measurements to compare the Δ*T*_m_ upon mutation (Table 2; Supplementary Figure S10). In the CD measurements, we observed that some mutants, such as L-D92A and H-D96A in 7C6, displayed larger error bars (±1.7°C). While differences in *T*_m_ values might seem insignificant, the slopes of the CD profiles in Supplementary Figure S10 could offer biophysical insights. For instance, although the Δ*T*_m_ value of L-D92A is only 0.3°C, a seemingly negligible difference from the WT, its slope increases more rapidly than the WT. This implies that the mutant unfolds faster than the WT upon exposure to increasing temperatures. Therefore, despite the need for caution, the subtle variations in *T*_m_ observed in this study could provide valuable insights into the affinity-stability trade-offs of the antibodies.

**FIGURE 7:**
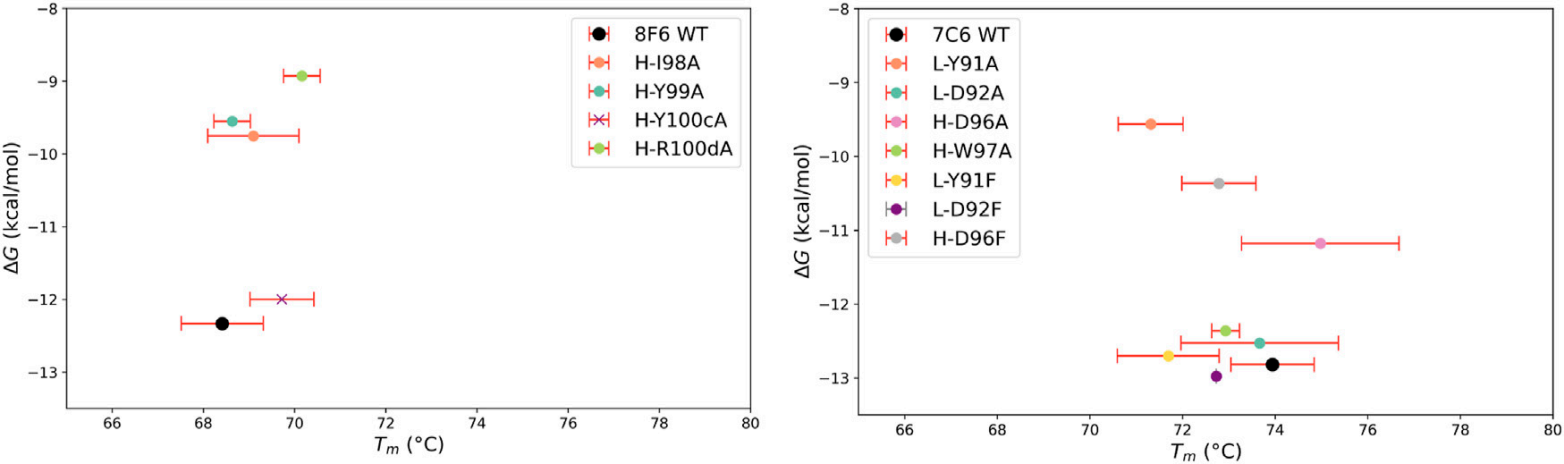
Relationship between the molar Gibbs free energy (Δ*G*) and thermal stability (*T*_m_). Δ*G* is obtained from the equilibrium dissociation constant, *K*_D_ using the following equation: Δ*G* = −*RT*ln(1/*K*_D_), where *R* is gas constant (0.0019872 kcal/mol·K) and *T* is temperature in Kelvin (298.15 K). Thermal stability is represented by melting temperature (*T*_m_). Error bars are shown for *T*_m_ calculated from three independent measurements for all antibodies except 7C6 L-D92F due to insufficient protein quantity. This is shown by gray error bar. Since kinetic parameters were not obtained for 8F6 H-Y100cA, the corresponding Δ*G* value does not directly reflect the effect on free energy for this mutant. To point out this discrepancy we marked this mutant with X.

The thermal stability results offer revealing insights when correlated with the nature of the amino acid pairs, specifically their relative hydropathy. Figure 7 illustrates the relationship between molar Gibbs free energy (Δ*G*) and stability, highlighting the intricate interplay between affinity-stability trade-offs. For hydrophobic-hydrophobic pairs found in CDR-H3 of both antibodies, residues H-D96 in 7C6, as well as H-I98/H-Y99 and H-Y100c/H-R100d in 8F6, exhibited an increase in *T*_m_, with a less favorable Δ*G*. This implies that the mutations have improved the thermal stability of the antibodies; however, this enhancement comes at the expense of an energetically less favorable binding reaction, resulting in a decrease in affinity. An exception among the hydrophobic-hydrophobic pairs was observed with H-W97 in 7C6. An alanine mutation in this residue led to a decrease in *T*_m_ (Δ*T*_m_ = −1.0°C), but did not significantly affect binding affinity (Table 2). Similar to a Tyr residue, a Trp residue seems to have a unique function; it contributes to aromatic interactions, acts as a hydrogen bond donor, possesses a large hydrophobic surface, and can shield delicate hydrogen bonds from water (Samanta et al., 2000). In contrast, in the case of the hydrophobic-hydrophilic pair within 7C6’s CDR-L3 (L-Y91/L-D92), a less favorable Δ*G* was observed alongside a decrease in *T*_m_. This suggests that the mutation has resulted in an energetically less favorable binding interaction, consequently leading to diminished binding affinity and a decrease in thermal stability. Notably, the negative Δ*G* associated with our predicted Phe mutant of 7C6 L-D92 suggests that this hydrophilic position is well-suited to accommodate the mutation and promotes an energetically favorable binding reaction. Among the identified hotspot pairs, the residues 7C6 L-Y91 and H-D96, along with 8F6 H-I98, H-Y100c and H-R100d, had a notable impact on *T*_m_. The marginal effect of 8F6 H-Y99A on *T*_m_ corroborates our hypothesis about the dual role of Tyr, as evidenced by our pattern analysis.

Our computational analysis and experimental measurements suggest that relative hydropathy influences the trend in thermal stability, whether increasing or decreasing (Figure 4; Table 2), while the IMGT-defined hydropathy highlights the importance of a residue’s contribution to stability (Figures 2, 4). This was particularly observed with the dual nature of Tyr (8F6 H-Y99). Recognizing the importance of both definitions provides a better understanding of the factors determining stability. Therefore, this study contributes to laying the groundwork for further exploration into the dual nature of Tyr in antibody and protein research.

## 3 Discussion

This study aims to investigate the binding affinity and stability of anti-MVH neutralizing antibodies, with the objective of exploring a potential correlation between binding affinity and stability. For this purpose, we proposed a hypothesis that high-affinity antibodies with differences in stability could provide valuable insights for our research objective. The physicochemical analysis revealed that antibodies 7C6 and 8F6 exhibited rapid association and slow dissociation with MVH, indicating high binding affinity (<1 nM). We focused on these two antibodies, which showed a significant difference in thermal stability (Δ*T*_m_, ∼6°C). Since no antibody crystal structure was available at the time of writing, homology modelling and knowledge-based local docking were performed to generate the apo (antibody) and holo (complex) forms, respectively.

Modeling antibody structures remains challenging, especially when the CDR-H3 extends beyond the average length (i.e., > 13–14 residues). While the modeling accuracy for non-CDR-H3 sections of antibodies is often satisfactory, even the state-of-the-art deep learning methods still struggle with CDR-H3 conformation predictions. On average, these predictions often deviate by more than 2.0 Å in backbone RMSD from crystal structures (Ruffolo et al., 2023). Such a 2 Å variance in backbone conformations is significant; even minor discrepancies (<1.0 Å) in backbone configurations can substantially alter the energy landscape of protein-protein interactions (Kuroda and Gray, 2016). Consequently, computer-guided affinity maturation studies without antibody crystal structures are scarce. A standout example is the work by Cannon et al. They integrated experiments with computational modeling to guide the affinity maturation of an antibody targeting an antigen (Cannon et al., 2019). Mutagenesis experiments were used to validate docking models and pinpoint the potential binding modes of the antibody-antigen complex. This was succeeded by re-docking of the complex and further design calculations based on the predicted model complex.

In our study, we sought to improve computational modeling accuracy by performing MD simulations immediately after modeling the antibody and docking it with the antigen. Within MD simulations, model structures undergo adjustments by interacting with the surrounding environment, including explicit water molecules. Based on these wholly computational outcomes, we were able to identify hotspots in the antibody-antigen interactions, a finding that our *in vitro* mutagenesis experiments subsequently validated. While the accuracy of ΔΔ*G* calculations by FoldX may be influenced by the quality of the input structures (Buß et al., 2018), our study’s strength lies in the experimental validations that corroborate our computational predictions. Although crystal structures of the complexes between MVH and the anti-MVH antibodies would offer valuable insights into molecular-level interactions, our study suggests that knowledge-based rigid-body docking simulations, followed by explicit solvent MD simulations, could serve as an effective alternative for exploring these interactions.

In protein engineering, the defined hotspots are a subset of residues composed of high affinity residues surrounded by low affinity residues as O-ring structure (Bogan and Thorn, 1998; Soga et al., 2010; Akiba and Tsumoto, 2015). We proposed a novel high and low ΔΔ*G* pattern that appears to effectively recognize these hotspots as a subset of two partner residues or pair. This pattern aided in identifying the hotspots responsible for significant loss in binding affinity for both the 7C6 and 8F6 antibodies. Through our investigation of high-affinity anti-MVH antibodies, we suggested a potential relationship between affinity and stability, which may offer insights into their trade-offs. We noted two distinct types of pairs based on their relative hydropathy: a) hydrophobic-hydrophilic and b) hydrophobic-hydrophobic. While the former type tended to show a decrease in stability along with a loss in binding affinity, the latter type seemed to maintain or increase stability despite a decrease in affinity.

In general, CDR-H3 is primarily responsible for antigen recognition and binding. However, it is intriguing to note that the highest affinity antibody, 7C6, possesses a shorter CDR-H3 (consisting of only 7 residues) compared to the other anti-MVH antibodies (2F4 and 10B5 with CDR-H3 of 12 residues, and 8F6 with CDR-H3 of 13 residues). This disparity in CDR-H3 length may explain why the CDR-H3 of 7C6 acts as a secondary hotspot.

A comparison of MVH binding to its receptors and antibodies in Supplementary Figure S11 shows that the binding site of 7C6 is predicted to be located within the region composed of amino acid residues 190–200, which is part of the immunodominant epitope (amino acids: 190–200 and 571–579). This epitope has been identified as a recognition site for mAb BH26, which inhibits the binding of approximately 60% of human serum antibodies in vaccinees and individuals recovering from measles (Ertl et al., 2003; Tahara et al., 2016). On the other hand, the predicted binding site of 8F6 lies within amino acids 505–552, which corresponds to the receptor binding epitope (residues 187, 190, 483, and 505–552). Additionally, residues within the CDR-H3 of 8F6 were found to interact with R533, which is part of a conserved neutralizing epitope (residues F483, D505, R533, Y541, and Y543). While there was a clear correlation between docking score and binding affinity, we also observed that the docking pose correlates with the inhibition capabilities of the anti-MVH antibodies. Supplementary Figure S11B illustrates the footprints of both receptors and the antibody on MVH. Although the overall binding sites seem similar, the CDR-H3 of 8F6, containing a hotspot (H-R100), is located near the hydrophobic pocket within the β4-β5 groove, a region implicated in receptor binding (Zhang et al., 2013). In contrast, a hotspot of 7C6 (L-Y91), experimentally identified in this study, is positioned in a region more distal from the hydrophobic pocket (Supplementary Figure S11B). This difference in the location of hotspots may account for the lower neutralizing capability of 7C6 compared to 8F6, as reported by Sato and colleagues (Sato et al., 2018), despite having higher affinity among the anti-MVH antibodies (Supplementary Figure S11A). Mutations at these conserved neutralizing epitope residues have been shown to facilitate immune escape from neutralization by the monoclonal antibody 2F4 (Santiago et al., 2010; Tahara et al., 2013). Although a co-crystal structure is currently unavailable, this study suggested the plausible binding mode of high-affinity antibodies to MVH (Supplementary Figure S11). Consequently, these findings open up avenues for further research on anti-MVH antibodies, providing valuable insights into their development.

Thus, this study highlighted the importance of a balance between hydrophobic and hydrophilic residues for achieving high affinity and stability in anti-MVH antibodies (Supplementary Figure S9). These findings pave the way for computational design strategies aimed at enhancing the affinity and stability of low-affinity anti-MVH antibodies, such as 2F4 and 10B5, in future research endeavors. When applying our approach to analyze the low-affinity anti-MVH antibodies, we identified residues that form pairs with Gly (Supplementary Figure S12). The absence of a side chain in one of the paired residues in 2F4 and 10B5 may contribute to their low affinity (Table 1). While Gly is known to play important roles in conformational flexibility, its specific influence on affinity, stability, and neutralization requires further investigation. Additionally, the pairs identified for 2F4 and 10B5 exhibit a hydrophobic gradient, suggesting that hydrophobic-hydrophilic combinations are relatively uncommon.

In this study, the combination of high-low ΔΔ*G* pattern and relative hydropathy analysis exhibit computational promise for addressing challenges related to the trade-offs between affinity and stability in antibody research. By training AI models with this pattern-driven analysis of antibodies, it may be possible to mitigate the need for large-scale experimental data. Therefore, it is essential to validate this pattern on additional antibodies targeting a range of antigens, in order to drive advancements in the field of antibody research facilitated by computational methods.

## 4 Methods

### 4.1 Antibody homology modeling and docking to antigen

The RosettaAntibody protocol (Weitzner et al., 2017) in Rosetta (Leman et al., 2020) was used to generate three-dimensional structure of the antibody Fv. To ensure comprehensive analysis, we generated 2000 structures for the top scored grafted model and 200 structures for the other grafted models. This enabled us to select the top-scoring model as a representative of the Fv structure among a wide variation of models.

At the time of our analysis, seven crystal structures of the MVH antigen were available in PDB, two of which were in the apo form, and the remaining structures were in complex with receptors. To identify the most suitable structure for docking, we selected the best resolution structure available (PDB: 2ZB6, 2.6 Å). Using Chimera v1.16 (Pettersen et al., 2004), we manually constructed a putative antigen-antibody complex. Subsequently, we employed the SnugDock protocol to perform a flexible backbone local docking, generating 1,000 poses of the anticipated antigen-antibody complex (Sircar and Gray, 2010).

### 4.2 Molecular dynamics simulations

The input structure for MD simulation were first modeled using Modeller 10.0 (Webb and Sali, 2016) for repairing the missing residues of MVH and constructing the constant regions of Fab. Then MD simulations were conducted using GROMACS 2022.4 (Berendsen et al., 1995; Lindahl et al., 2001; Abraham et al., 2015) with the CHARMM36m force field (Huang et al., 2017) to explore the behavior of the docked models. To solvate the system, TIP3P water (Madura et al., 1983) was used to fill a cubic box, and the protein was placed at the center with a 10 Å minimum distance to the box edge, while periodic boundary conditions were applied. Additional Na^+^ or Cl^−^ ions were introduced to neutralize the protein charge and simulate a salt solution with a concentration of 0.15 M. Each system was energy-minimized for 5,000 steps with the steepest descent algorithm and equilibrated with position restraints of protein heavy atoms and NVT ensemble, where the temperature was increased from 50 to 298 K during 200 ps. Further non-restrained simulations were performed with the NPT ensemble at 298 K for 240 ns. The time step was set to 2 fs throughout the simulations. A cutoff distance of 12 A was used for Coulomb and van der Waals interactions. Long-range electrostatic interactions were evaluated by means of the particle mesh Ewald method (Darden et al., 1993). Covalent bonds involving hydrogen atoms were constrained by the LINCS algorithm (Hess et al., 1997). A snapshot was saved every 100 ps. We performed three independent production runs with distinct initial velocities. All subsequent analyses were conducted using the GROMACS package.

### 4.3 *In silico* alanine scanning and mutational design

FoldX (v4) *AlaScan* command was utilized to identify potential hotspots on the antibody (Schymkowitz et al., 2005). Both apo and holo models underwent alanine scanning to predict the effect of mutations on binding with the antigen and antibody. We obtained difference in the free energy, or ΔΔ*G* values for both apo (ΔΔ*G*_apo_) and holo (ΔΔ*G*_holo_) forms in kcal/mol from each analysis and averaged them for each position (ΔΔ*G*).

$${\Delta \Delta G}_{\text{holo}}={\Delta G}_{Mut\_holo}-{\Delta G}_{WT\_holo}$$

$${\Delta \Delta G}_{\text{apo}}={\Delta G}_{Mut\_apo}-{\Delta G}_{WT\_apo}$$

$$\Delta \Delta G=average\left({\Delta \Delta G}_{\text{holo}}+{\Delta \Delta G}_{\text{apo}}\right)$$

Using ΔΔ*G* from Ala scan as a reference, we performed mutational design. Mutations for positions with low ΔΔ*G* were predicted using FoldX *BuildModel* command (van Durme et al., 2011), while positions with high ΔΔ*G* were predicted using the Rosetta’s Cartesian_ddg application (Kellogg et al., 2011; Park et al., 2016). A cut-off value of −1 kcal/mol was used for selecting mutants for *in vitro* mutagenesis study.

### 4.4 Spatial aggregation propensity (SAP)

The SAP (Chennamsetty et al., 2009) algorithm was used to predict relative hydropathy with an in-house CHARMM-based script (Brooks et al., 2009). The SAP was calculated on the holo form and score for each atom within a 10 Å radius was calculated by this algorithm. As a result, a residue wise score was obtained in an output file. The maximum (positive) and minimum (negative) values on the SAP scale indicate hydrophobicity and hydrophilicity of the scale.

### 4.5 Cloning, expression, and purification of antibodies

The DNA sequences encoding the heavy and light chains of the Fab antibodies were codon-optimized and synthesized by Integrated DNA Technologies, Inc. They were subcloned into separate pcDNA3.4 vectors (Thermo Fisher Scientific), with a His_6_ tag fused to the C-terminus of the heavy chains by HiFi DNA assembly (NEB). The DNA of the mutants was prepared by site-directed mutagenesis PCR using the KOD -Plus- Mutagenesis Kit (TOYOBO). The protocol was slightly modified, as we used KOD One PCR Master Mix (TOYOBO) instead of KOD -Plus-. The Fab antibodies were expressed in ExpiCHO cells (Thermo Fisher Scientific) following the max titer protocol for 8F6 antibody, and in Expi293 cells (Thermo Fisher Scientific) following the manufacturer’s standard protocol for rest of the antibodies (Fang et al., 2017; Jain et al., 2017). The cells were cultured by rotating at 125 rpm at 37°C and 8% CO_2_ for 5 days for Expi293 cells, and at 32°C and 5% CO_2_ for 13 days for ExpiCHO cells after co-transfecting the cells with 13 µg of the heavy and light chain encoding plasmids. The culture supernatant was collected by centrifugation for 10 min at 5,000 g, dialyzed with a solution of 20 mM Tris-HCl (pH 8), 500 mM NaCl, 5 mM imidazole (binding buffer), and filtered with 0.8 μm filters (Advantec). It was loaded onto a Ni-NTA Agarose resin (Qiagen) equilibrated with binding buffer for immobilized metal affinity chromatography. After washing the resin with 10 mL of binding buffer, the protein was eluted with the buffers containing increasing concentrations of imidazole. The antibodies were obtained after further purification by size-exclusion chromatography (SEC) using HiLoad 26/600 Superdex 75 pg column (Cytiva) at 4°C equilibrated with phosphate-buffered saline (PBS) pH 7.4. The concentration of the proteins was calculated from the molecular weights and molar extinction coefficients (cm^−1^M^−1^) calculated from the amino acid sequences using ProtParam Tool (ExPASy) (Gasteiger et al., 2005) and the absorbance at 280 nm obtained on NanodropOne (Thermo Fisher Scientific).

### 4.6 Cloning, expression, and purification of antigen hemagglutinin

The pHLsec-vector plasmid with the MVH head domain (amino acid residues 149–617) was transiently transfected into 293S GnTI (−) cells (Hashiguchi et al., 2007). The cells were cultured for 4 days after transfection at 37°C and 5% CO_2_. The culture supernatant was collected by centrifugation at 7,000 rpm for 20 min at 4°C and filtration. The collected supernatant was purified with a complete His-Tag Purification Resin (Roche, Cat# 5893682001) affinity column after equilibration with 50 mM NaH_2_PO_4_・2H_2_O, 150 mM NaCl, and 10 mM imidazole. The resin capturing the head domain of MVH was washed with 25 mM NaH_2_PO_4_・2H_2_O, 75 mM NaCl, and 5 mM imidazole, and subsequently, the protein was eluted with the buffers containing increasing concentrations of imidazole. The head domain of MVH was obtained after further purification by SEC using Superdex 200 Increase 10/300 GL column (Cytiva) equilibrated with PBS. The concentration of the head domain of MVH was also confirmed following the same protocol as above.

### 4.7 Surface plasmon resonance (SPR)

The kinetic parameters of the antigen-antibody binding were determined by SPR using Biacore T200 instrument (Cytiva). The antigen hemagglutinin was immobilized on a CM5 sensor chip (Cytiva) at around 500 resonance units following the manufacturer’s amine coupling protocol. The Fabs were injected into the sensor chip at a flow rate of 30 μL/min at 25°C. The binding response at the following concentrations 62.5, 125, 250, 500, and 1,000 nM for 2F4 and 10B5, and 1.25, 2.5, 5, 10, and 20 nM for 7C6 and 8F6 wild type antibodies were used for the experiment. The concentrations used for 7C6 mutants except L-Y91A, were 1.25, 2.5, 5, 10, and 20 nM. For 7C6 L-Y91A mutation we used the following dilutions 6.25, 12.5, 25, 50, and 100 nM. For 8F6, two mutants H-D96A and H-I98A, used the following concentrations 1.25, 2.5, 5, 10, and 20 nM, like wild type antibody. For, 8F6 mutants H-Y99A, H-Y100 cA and H-R100dA, the following concentrations 190, 380, 750, 1,500, 3,000 nM; 250, 500, 1,000, 2000 and 4,000 nM; and 62.5, 125, 250, 500, and 1,000 nM, were used respectively. The association and dissociation time for wild 2F4, 10B5 and 8F6 mutant H-Y100 cA were 120 s and 600 s, respectively. For the rest of the Fabs including wild type and mutants for 7C6 and 8F6, a 120 s of association and 1,200 s of dissociation time were used in the experiment. The assays were carried out in HBS-T buffer (10 mM HEPES pH 7.5, 150 mM NaCl and 0.005% [v/v] Tween 20 surfactant). Biacore Insight Evaluation Software (Cytiva) was used to calculate the binding parameters.

### 4.8 Differential scanning calorimetry (DSC)

The thermal stability of the wild type antibodies was measured by DSC using MicroCal PEAQ-DSC (Malvern; Worcestershire, UK). The Fab samples (1 mg/mL) were prepared in PBS. At a scanning rate of 1°C/min the samples were heated from 20°C to 110°C. The data was fitted by non-two-state model using MicroCal PEAQ-DSC software (Malvern).

### 4.9 Circular dichroism (CD) measurements

The Fab’s CD profile and thermal stability were measured using a JASCO J-820 spectropolarimeter. The CD spectra were obtained from 260 to 200 nm using a 1 mm quartz cuvette with a protein sample of 0.1 mg/mL in PBS. Each sample was measured five times with a 1 nm bandwidth. To analyze the protein denaturation profile, the thermal stability was measured at lower concentrations under the same buffer conditions and with three repetitions, at 1°C intervals from 30°C to 90°C and at a speed of 0.1°C/min, at 218 nm and 215 nm ellipticity for 7C6 and 8F6 wild type and mutants, respectively. The *T*_m_ was determined by fitting the ellipticity data against temperature using nonlinear least squares curve fitting that followed the below logistic function equation, followed by sigmoid curve fitting in Python 3.0 (Rossant, 2018) to obtain the fitted molar ellipticity and temperature values.

$$f_{L,m,k,x0}\left(x\right)=\frac{L}{1+\exp\left(-k\left(x-x0\right)\right)}+m$$

Where, L, m, k and x0 are the vector parameters for optimization of the fitting. For better visualization of the *T*_m_ measurements, we represent the derivative of the fitted data.

## Data Availability

The MD trajectories and the docking model structures have been submitted to the Biological Structure Model Archive (BSM-Arc) under BSM-ID BSM000047 [https://bsma.pdbj.org/entry/47] (Bekker et al., 2020).
