# Supplementary material for: Unveiling the affinity–stability relationship in anti-measles virus antibodies: a computational approach for hotspots prediction
Source: Front Mol Biosci. 2024 Mar 1;10:1302737. doi: 10.3389/fmolb.2023.1302737 (PMC10941800; doi:10.3389/fmolb.2023.1302737)
Supplement: Supplementary file 1 [file DataSheet1.PDF]

Table S1

Table S1. Germline gene usage of the antibodies in this study. IMGT/DomainGapAlign was used to identify the germline genes. This table also reports the percentage identity at the amino acid level for both the V-REGION and J-REGION of the four mouse anti-MVH virus antibodies.

	VH domain		VL domain	
	**IGHV (% identity)**	**IGHJ (% identity)**	**IG(K/L)V (% identity)**	**IG(K/L)J (% identity)**
**2F4**	IGHV5-17*01 (100)	IGHJ4*01 (100)	IGKV12-46*01 (100)	IGKJ1*01 (100)
**10B5**	IGHV1-9*01 (90.8)	IGHJ4*01 (100)	IGKV6-25*01 (96.8)	IGKJ2*01 (100)
**7C6**	IGHV1-5*01 (89.8)	IGHJ1*01 (100)	IGKV14-111*01 (95.8)	IGKJ2*01 (100)
**8F6**	IGHV1-4*02 (94.8)	IGHJ3*01 (100)	IGKV10-96*01 (93.7)	IGKJ1*01 (100)

Figure S1

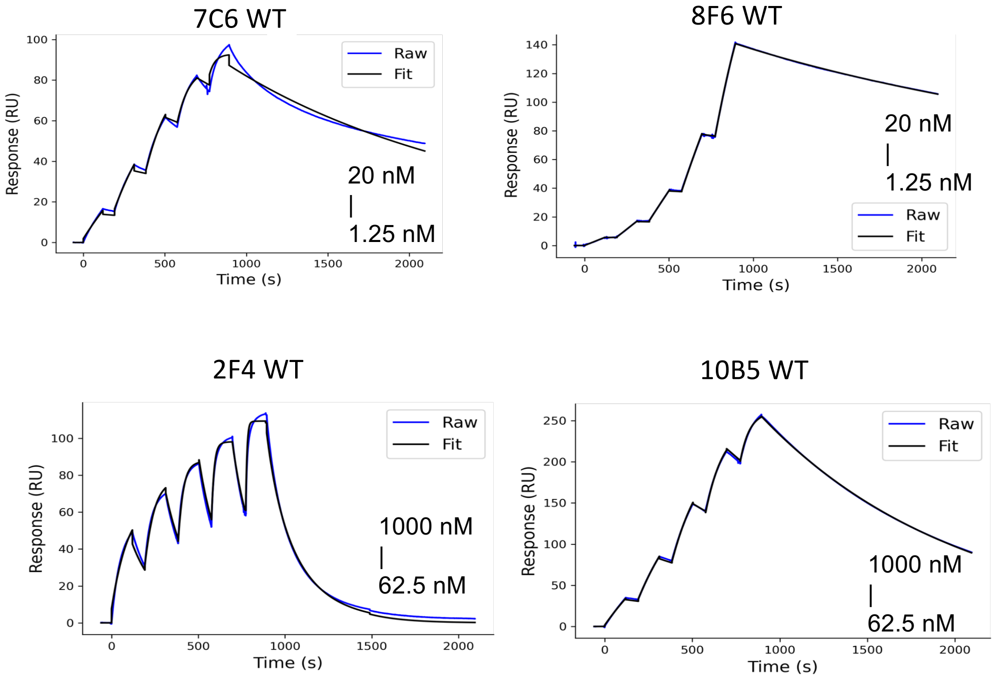

Figure S1. Physicochemical analysis of the wild type anti-MVH antibodies. Sensorgrams of anti-MVH wild type antibodies binding with hemagglutinin evaluated by SPR.

Figure S2

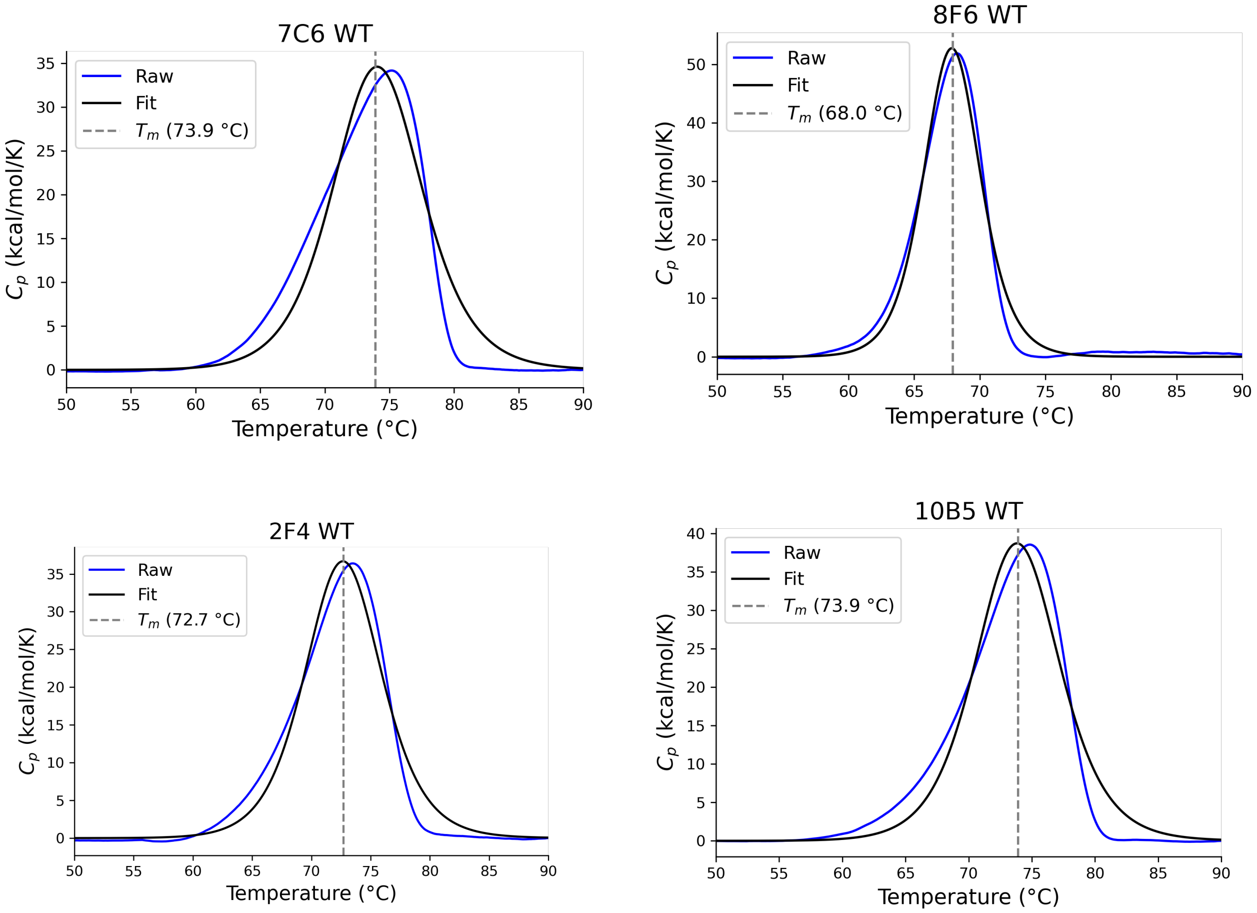

Figure S2. Physicochemical analysis of the wild type anti-MVH antibodies. Thermal stability of the wild type antibodies measured by DSC. The melting temperature (T_m) are presented.

Figure S3

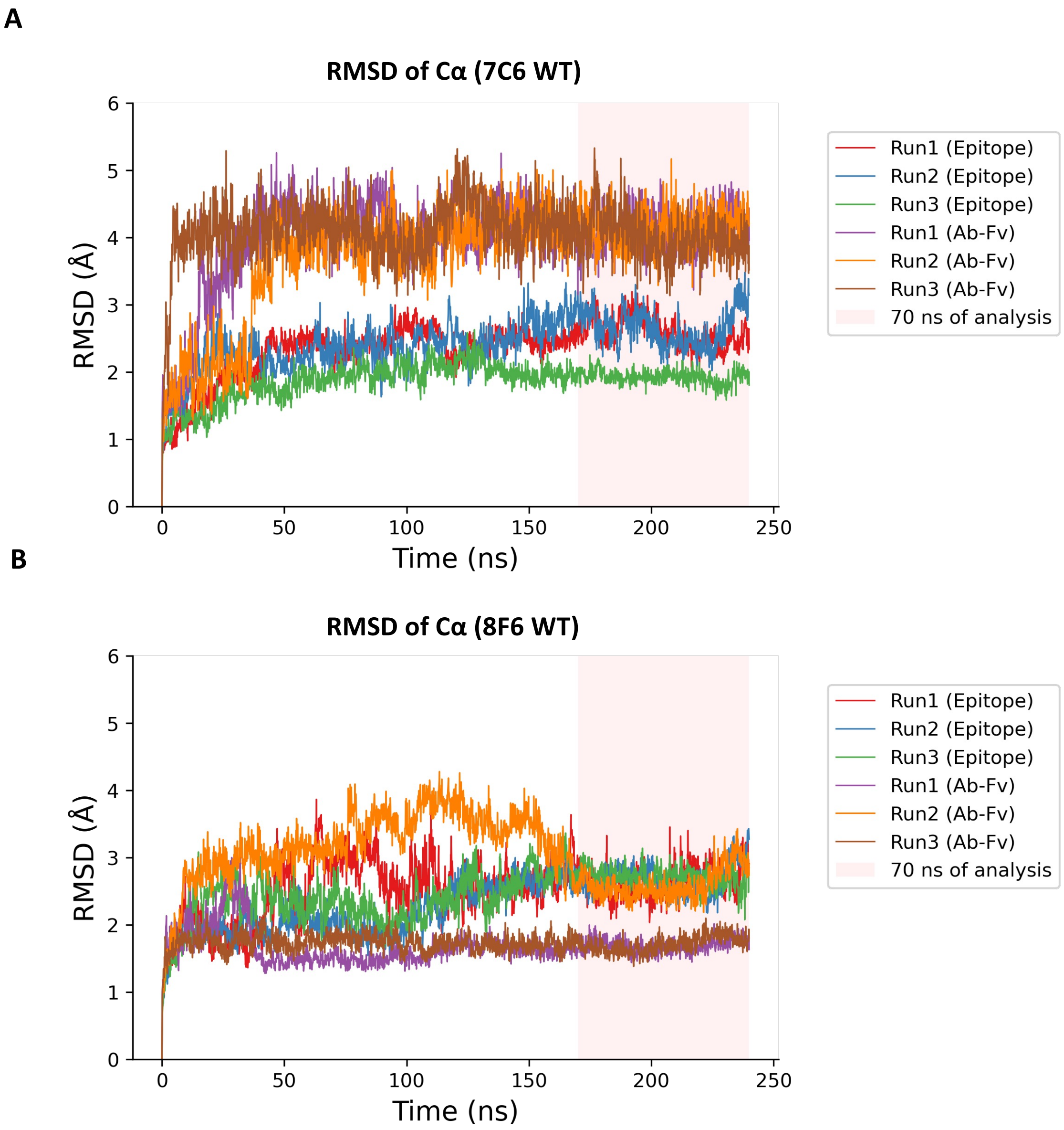

Figure S3. Quantitative analysis of MD simulations. (A) and (B) display of root mean square deviation (RMSD) of Cα atoms for epitope and variable region (Fv) of antibody (Ab) for 7C6 and 8F6 antibodies, respectively, across three independent runs. After achieving the convergence of the simulation, 70 ns of trajectories (shaded in pink) were selected for interaction analysis.

Figure S4

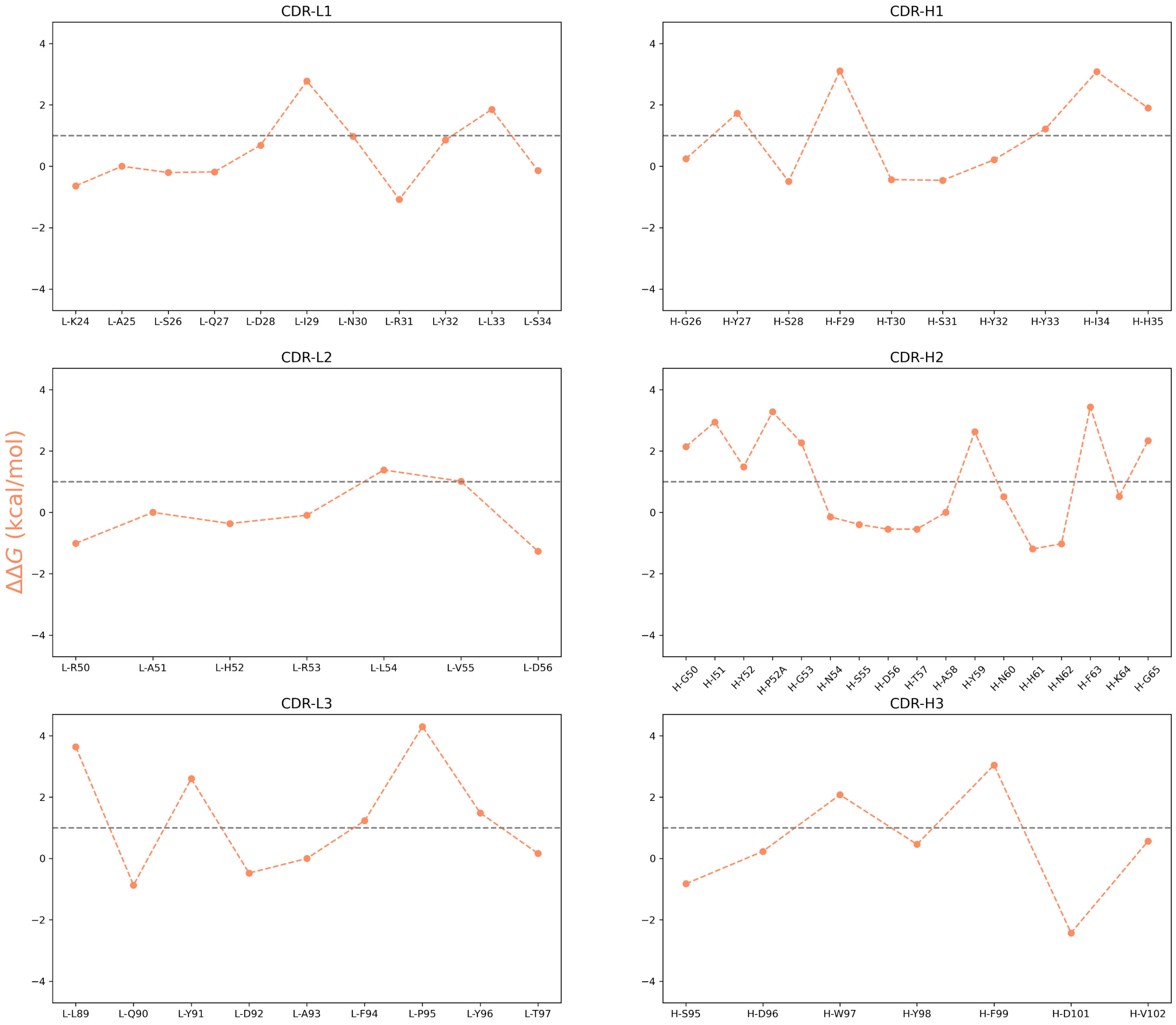

Figure S4. . *In silico* alanine scanning of CDRs of 7C6. Show the results of *in silico* alanine scanning using the FoldX AlaScan command. The results are depicted as an orange line. The $\Delta\Delta G$ cut-off = 1 kcal/mol is represented by dashed line.

Figure S5

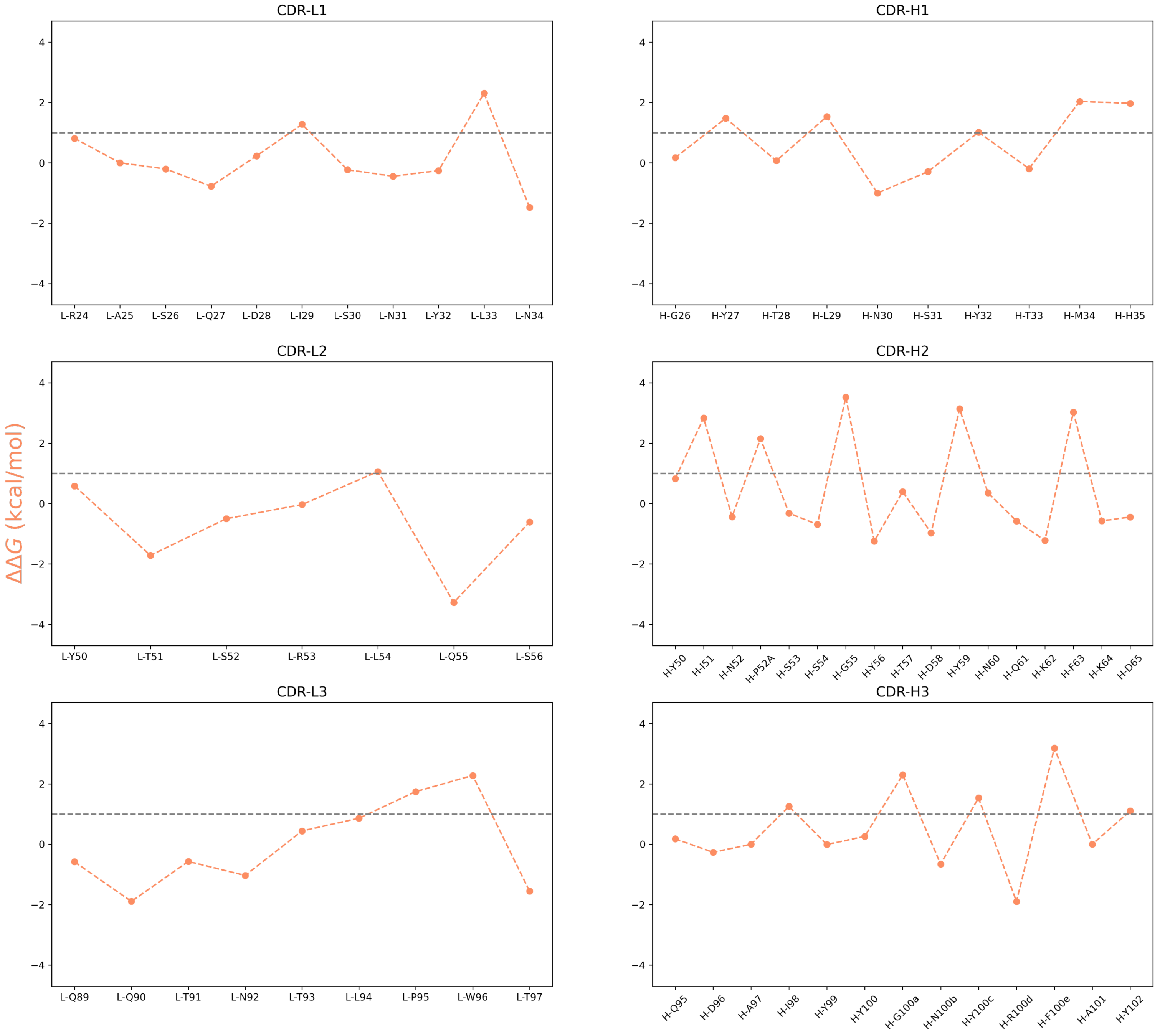

Figure S5. *In silico* alanine scanning of CDRs of 8F6. Show the results of *in silico* alanine scanning using the FoldX AlaScan command. The results are depicted as an orange line. The ΔΔG cut-off = 1 kcal/mol is represented by dashed line.

Figure S6

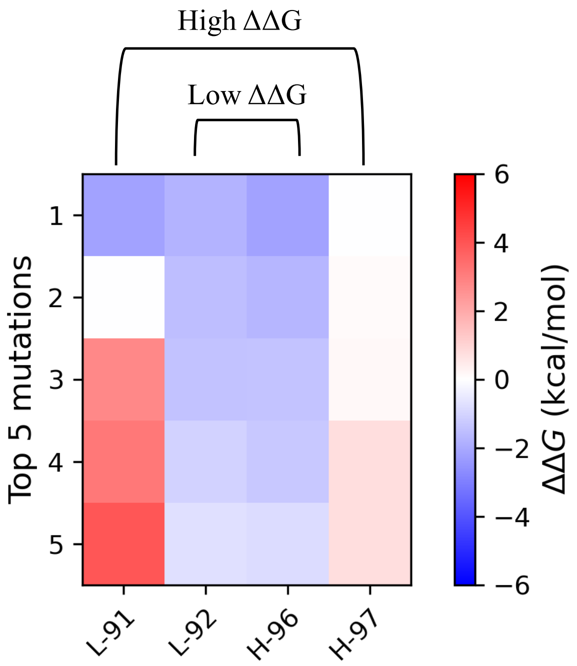

Figure S6. Heatmap of the top 5 predicted mutations for 7C6. The ΔΔ*G* values calculated by FoldX and Rosetta cartesian ddg are represented. Positions with high ΔΔ*G* and low ΔΔ*G* from Alascan are labeled. Rosetta and FoldX were used for high and low ΔΔ*G*-labeled positions, respectively. 5 predicted mutations are shown, where 1 in Y-axis suggests the best ΔΔ*G* for that position.

Figure S7

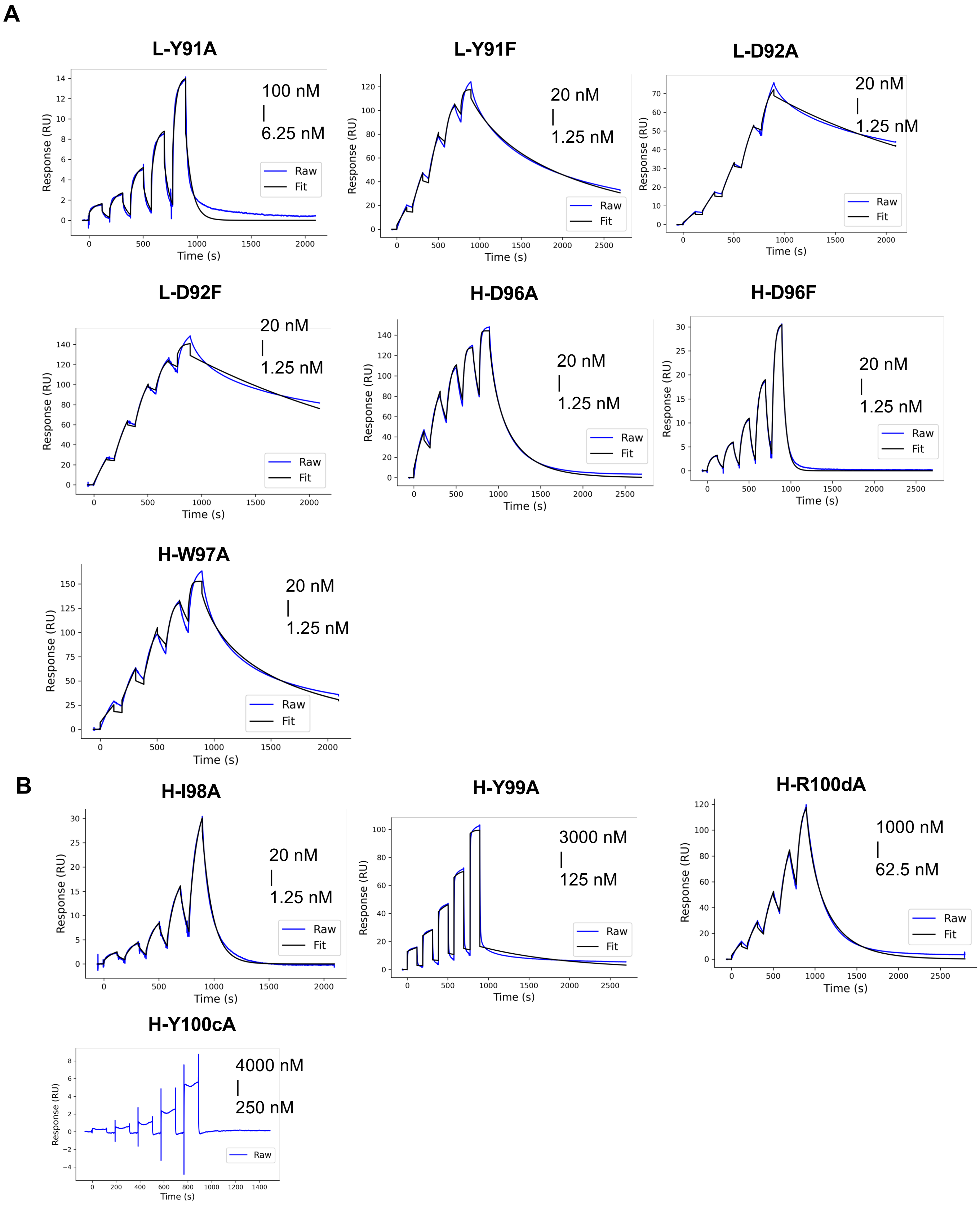

Figure S7. Physicochemical analysis of the mutants. (A) and (B) Sensorgrams of the mutant antibodies binding with hemagglutinin evaluated by SPR. The SPR results include 5 serial dilutions with the highest and lowest concentrations for each mutant. Raw data is presented for 8F6 H-Y100cA mutant as kinetic fitting was not applicable.

Figure S8

A

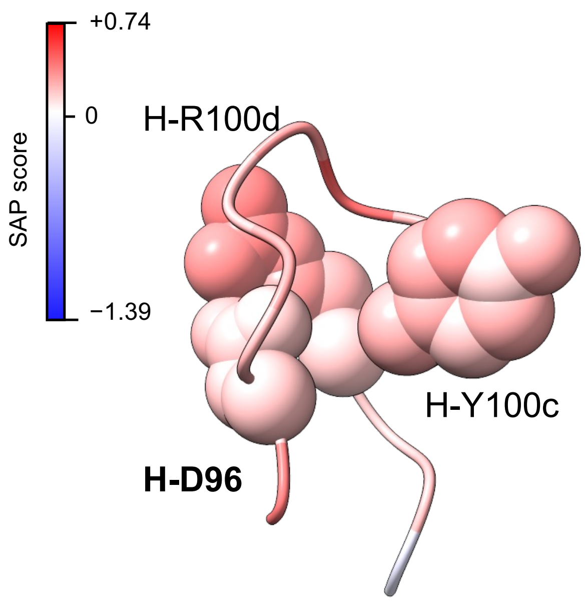

B

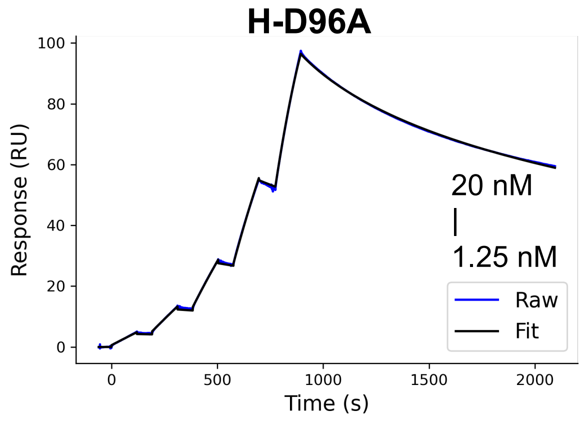

C

	k_{on} (×10^5 M^{-1}s^{-1})	k_{off} (×10^{-4} s^{-1})	K_D at 25 °C (nM)
**8F6 WT**	3.4 ± 2.9	2.8 ± 1.7	0.9 ± 0.2
H-D96A	6.8 ± 5.2	17.4 ± 21.9	1.9 ± 1.8

Figure S8. Validation of our protocol on a negative control. (A) shows residue H-D96, which is spatially near the hotspot pair H-Y100c/H-R100d in 8F6 CDR-H3. The residues are displayed as atoms in a sphere style, with color coding based on the SAP score. The corresponding SAP scale is also depicted in the image. (B) The SPR results include 5 serial dilutions with the highest and lowest concentrations for the Ala mutant. (C) kinetic parameters are reported.

Figure S9

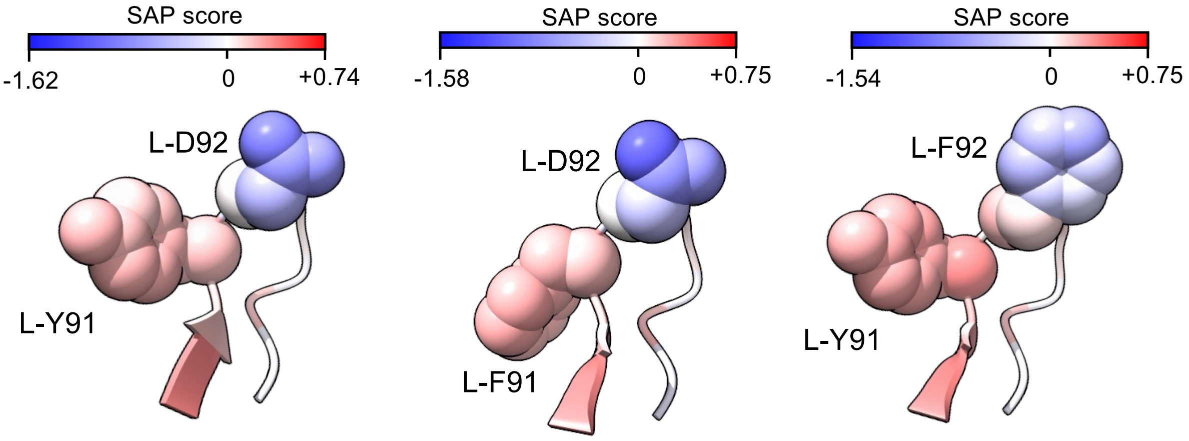

	7C6 WT	L-Y91F	L-D92F
Docking score (REU)	-41.0	-43.7	-42.2
Backbone RMSD (Å)	-	0.2	0.3
Affinity (nM)	0.4 ± 0.2	0.5 ± 0	0.3 ± 0
Stability (°C)	73.9 ± 0.9	71.7 ± 1.1	72.7 ± 0

Figure S9. Relative hydropathy of Phe mutants to pair 7C6 L-Y91/L-D92. The hotspot pair L-Y91/L-D92 is shown for the WT and mutants. The residues are displayed as atoms in a sphere style, with color coding based on the SAP score. The corresponding SAP scale is also depicted in the image. The reported data encompasses the docking score obtained through RosettaDock, the backbone RMSD of the CDR-L3 region calculated in UCSF Chimera, as well as affinity and stability measures.

Figure S10

A

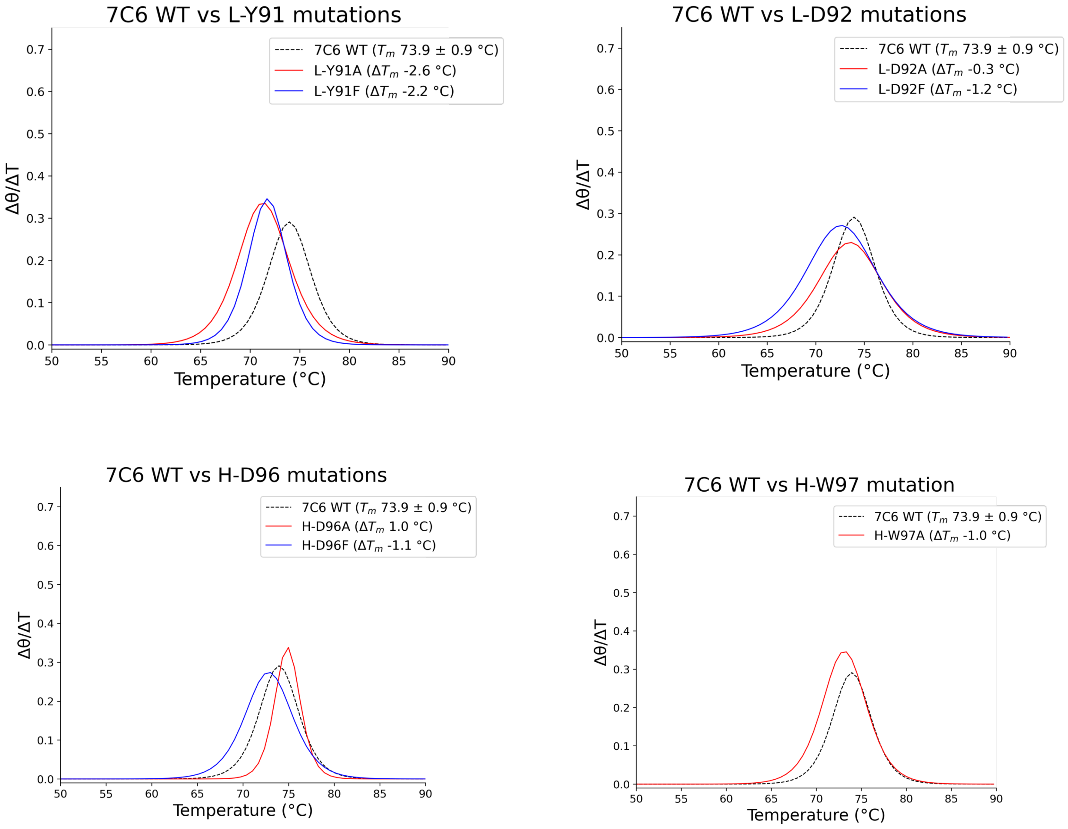

B

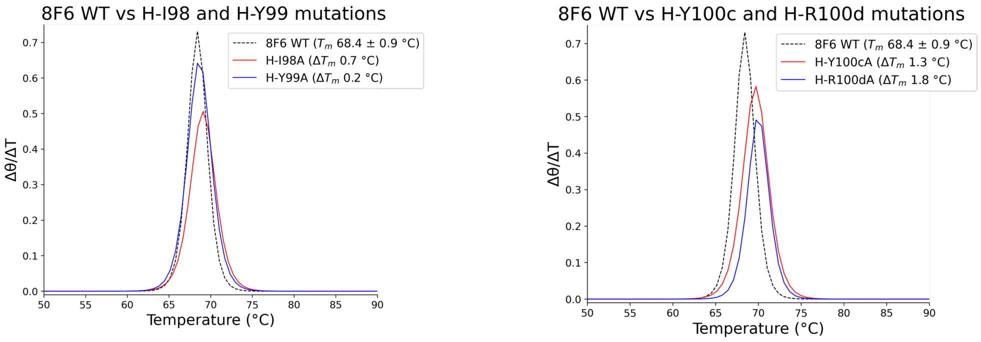

Figure S10. Thermal stability of the mutants. (A) and (B) demonstrate the thermal stability measured by CD for the 7C6 and 8F6 mutants, respectively. The CD profile for each mutant represents the average of three independent measurements, except for 7C6-LD92F, which was limited by the quantity of the protein available. The corresponding change in melting temperature (ΔT_m) for each mutant is also reported.

Figure S11

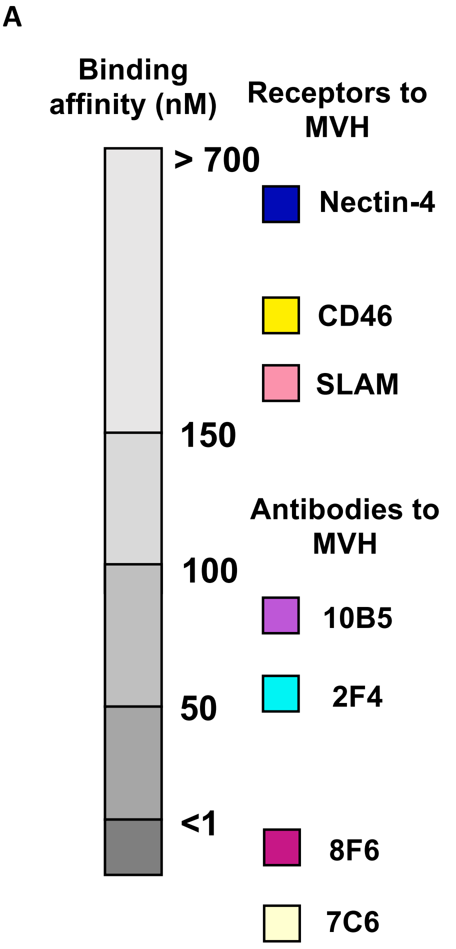

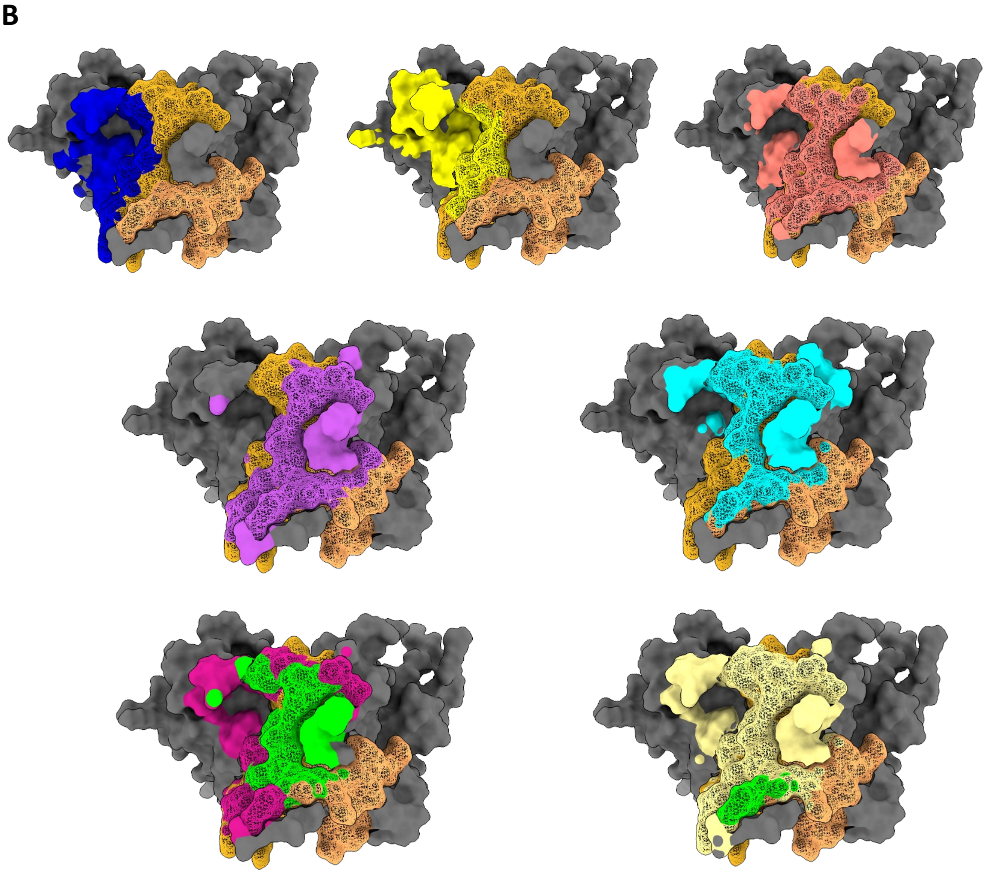

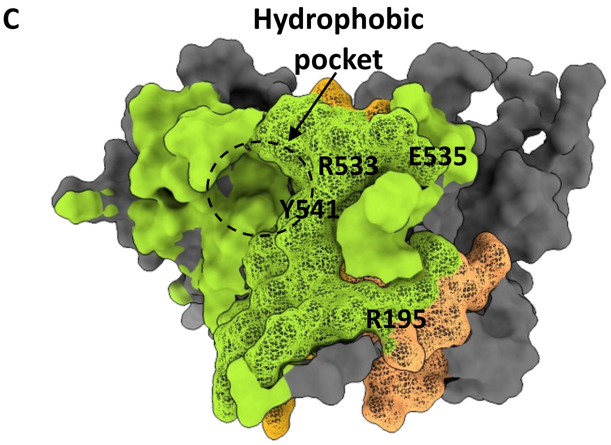

D

	Nectin-4	CD46	SLAM	2F4	8F6	7C6
R195			✓			✓
R533			✓	✓	✓	
E535					✓	
Y541	✓	✓		✓		

E

	10B5	2F4	8F6	7C6
Nectin-4	*	***	***	**
SLAM	*	**	***	***
NT2N	***	**	***	×

Figure S11. Comparison of MVH binding to its receptors and antibodies to unveil the effect of binding pose on virus inhibition. (A) categorizes the binding affinity of receptors Nectin-4, CD46 and SLAM (Tadokoro et al., 2020, Santiago et al., 2010) and antibodies to MVH. The receptors and antibodies are color-coded to represent the structure in panel (B). (B) Individual receptor and antibody footprints in MVH. The receptors studied included nectin-4 (PDB ID: 4GJT), CD46 (PDB ID: 3INB) and SLAM (PDB ID: 3ALZ). Alongside these, both low-affinity antibodies (10B5 and 2F4) and high-affinity antibodies (7C6 and 10B5) were considered, utilizing their best-scored docked models. The receptor/antibody binding on the MVH was defined by residues within 5 Å of the receptor/antibody in the individual complexes. The surface of the MVH is shaded as per (A). The green highlights indicate the contact points for CDR-H3 and CDR-L3 in 8F6 and 7C6, respectively (*see main text*). The epitope used in this study represented as a mesh-like surface. (C) Combined receptor and antibody footprints in MVH. The contact residues in the epitope are highlighted in green. Residues that were identified to be interacting in MD and are reported previously are labeled for 7C6, 8F6 and 2F4, respectively. (D) represents the interacting residues depicted in (C), comparing them with the hotspots identified in 7C6, 8F6, 2F4 (Tahara et al., 2016), as well as with the receptors (Zhang et al. 2013). The residue interactions are shown with tick marks. (E) Inhibition of virus spread in Vero/hSLAM, Vero/hNectin4 and NT2N cells, as reported by Sato and colleagues (Sato et al., 2018). The * represents the degree of inhibition of virus spread, where *** represents strong inhibition. '×' denotes failed inhibition.

Figure S12

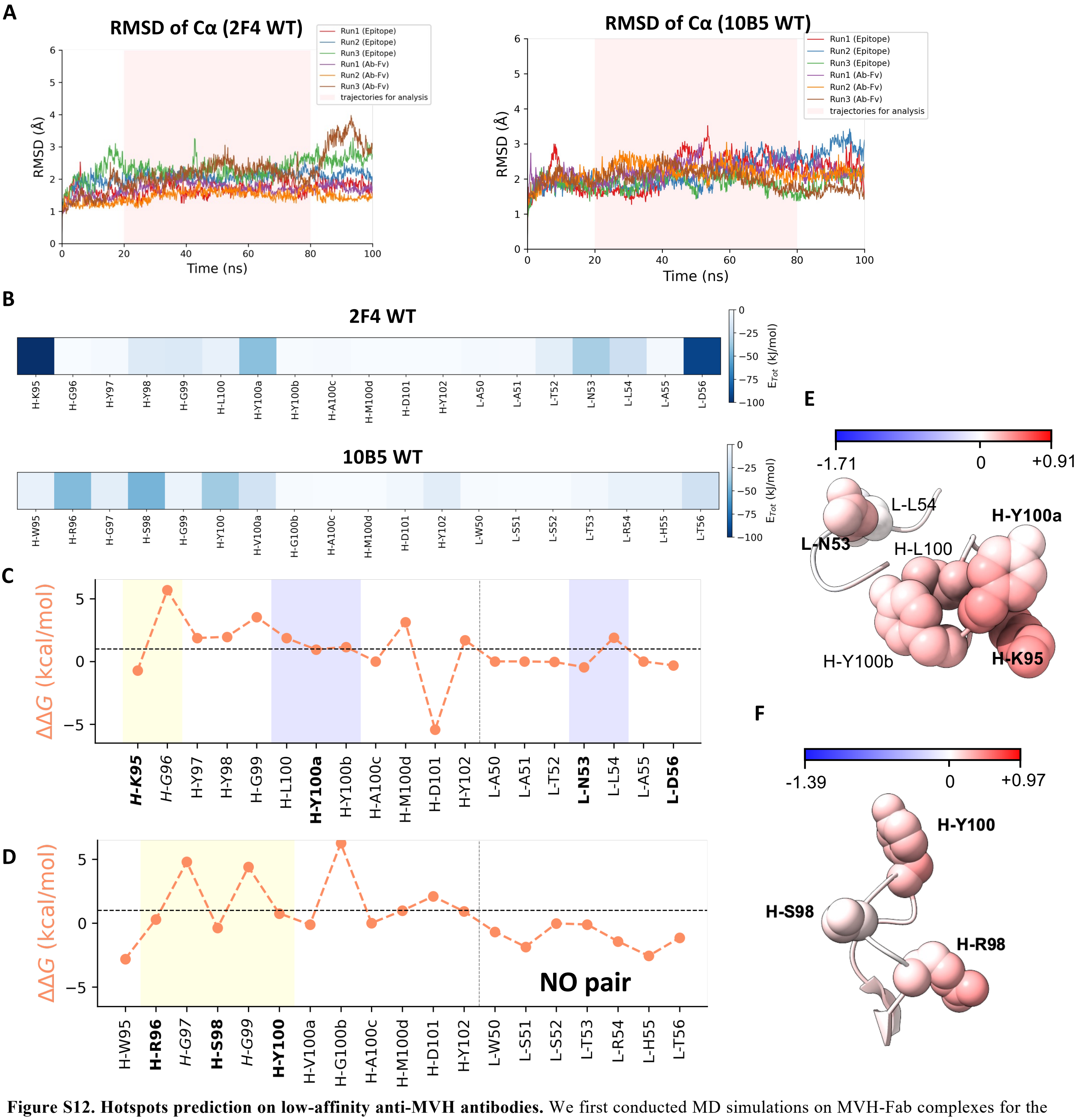

Figure S12. Hotspots prediction on low-affinity anti-MVH antibodies. We first conducted MD simulations on MVH-Fab complexes for the low-affinity anti-MVH antibodies. (A) depicts the RMSD of Cα atoms for the epitope and variable region (Fv) of the antibody (Ab) for 2F4 and 10B5 antibodies, respectively, across three independent runs. After achieving the convergence of the simulation, 60 ns of trajectories (shaded in pink) were selected for interaction analysis, as illustrated in (B). (B) displays the heatmaps of residue-wise E_{Tot} between the epitope and CDRs H3 and L2 of 2F4 and 10B5. The data shown represents the average of three independent MD simulations. The total non-bonded interaction energy (E_{Tot}) is expressed in kJ/mol. (C) and (D) show the results of *in silico* alanine scanning. The results are depicted as an orange line. The ΔΔG cut-off = 1 kcal/mol is represented by a dashed line. These plots highlight (in bold) the residues identified in (B) for 2F4 (illustrated in (E)) and 10B5 (illustrated in (F)). The plots feature shaded regions in both yellow and purple colors. The yellow highlights indicate the absence of a side chain in one of the paired residues, while the purple highlights denote the presence of side chains in the residue pair. (E) and (F) pair residues identified in (C) and (D) are displayed as atoms in a sphere style, with color coding based on the SAP score. The corresponding SAP scale used for both antibodies is also depicted in the image.

Supplementary References:

Santiago, C., Celma, M. L., Stehle, T., & Casasnovas, J. M. (2010). Structure of the measles virus hemagglutinin bound to the CD46 receptor. *Nature Structural & Molecular Biology*, *17*(1), 124–130. https://doi.org/10.1038/NSMB.1726.

Sato, Y., Watanabe, S., Fukuda, Y., Hashiguchi, T., Yanagi, Y., & Ohno, S. (2018). Cell-to-Cell Measles Virus Spread between Human Neurons Is Dependent on Hemagglutinin and Hyperfusogenic Fusion Protein. *Journal of Virology*, *92*(6). https://doi.org/10.1128/JVI.02166-17.

Tadokoro, T., Jahan, M. L., Ito, Y., Tahara, M., Chen, S., Imai, A., Sugimura, N., Yoshida, K., Saito, M., Ose, T., Hashiguchi, T., Takeda, M., Fukuhara, H., & Maenaka, K. (2020). Biophysical characterization and single-chain Fv construction of a neutralizing antibody to measles virus. *The FEBS Journal*, *287*(1), 145–159. https://doi.org/10.1111/FEBS.14991.

Zhang, X., Lu, G., Qi, J., Li, Y., He, Y., Xu, X., Shi, J., Zhang, C. W. H., Yan, J., & Gao, G. F. (2013). Structure of measles virus hemagglutinin bound to its epithelial receptor nectin-4. *Nature Structural & Molecular Biology*, *20*(1), 67–72. https://doi.org/10.1038/NSMB.2432.
